# 1,2,4-Oxadiazole-Bearing Pyrazoles as Metabolically Stable Modulators of Store-Operated Calcium Entry

**DOI:** 10.1021/acsmedchemlett.1c00034

**Published:** 2021-03-10

**Authors:** Silvio Aprile, Beatrice Riva, Irene Preet Bhela, Celia Cordero-Sanchez, Giulia Avino, Armando A. Genazzani, Marta Serafini, Tracey Pirali

**Affiliations:** † Department of Pharmaceutical Sciences, Università degli Studi del Piemonte Orientale, Largo Donegani 2, 28100 Novara, Italy; ‡ ChemICare S.r.l., Enne3, Corso Trieste 15/A, 28100 Novara, Italy; § Department of Pharmaceutical Sciences, Università degli Studi di Trieste, Via Giorgieri 1, 34127 Trieste, Italy

**Keywords:** Drug discovery and development, Store-Operated Calcium Entry, 1,2,4-Oxadiazole, Bioisosteric replacement, CRAC channels

## Abstract

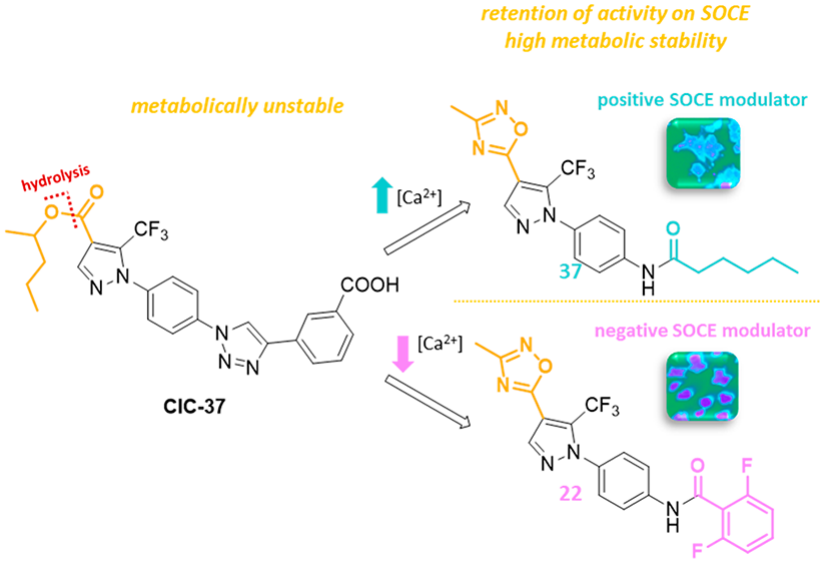

Store-operated calcium entry (SOCE) is a pivotal mechanism in calcium homeostasis, and, despite still being under investigation, its dysregulation is known to be associated with severe human disorders. SOCE modulators are therefore needed both as chemical probes and as therapeutic agents. While many small molecules have been described so far, their poor properties in terms of drug-likeness have limited their translation into the clinical practice. In this work, we describe the bioisosteric replacement of the ester moiety in pyrazole derivatives with a 1,2,4-oxadiazole ring as a means to afford a class of modulators with high metabolic stability. Moreover, among our derivatives, a compound able to increase the calcium entry was identified, further enriching the library of available SOCE activators.

The ubiquitous second messenger calcium is a central regulator of a plethora of physiological processes, spanning from fertilization of oocytes to cell death, mobility, secretion, and gene expression. Infinitesimal oscillations of this ion concentration in the different cell compartments control a multitude of processes. This is the reason why every cell maintains a huge gradient between the plasma membrane or the intracellular calcium repository and the cytoplasm, with various pumps and exchangers in charge of finely regulating its levels.^1,2^ A central mechanism in calcium homeostasis is represented by the store-operated calcium entry (SOCE), *i.e.*, the influx of calcium across the plasma membrane activated by the depletion of this ion in the endoplasmic reticulum (ER).^3^ SOCE is mediated mainly, although not exclusively, by two protein families: STIM and Orai. STIM1 and its homologue STIM2 are single-span membrane proteins residing on the ER membrane and act as sensors of calcium levels. Orai are a family of proteins (Orai1, Orai2 and Orai3) that form the ion channels on the plasma membrane.^4−6^ When a depletion of calcium from the ER stores occurs, STIM undergoes a conformational change leading to self-association into puncta and migration near the plasma membrane. There, interaction with Orai promotes the opening of the channel and the subsequent influx of calcium. The empty ER stores are then refilled by the sarco-endoplasmic reticulum calcium ATPase (SERCA) pump.^7,8^ Due to its prominent role in encoding calcium signals, SOCE is implicated in several human disorders, including cancer,^9,10^ inflammatory bowel disease,^11^ allergy,^12^ and acute pancreatitis.^13,14^ Therefore, it is not surprising that this cellular pathway has become a promising target for the therapeutic treatment of several pathological conditions.

Over the years, many small molecules^15−18^ able to modulate SOCE have been reported. The earliest modulators are SKF-96365,^19,20^ 2-APB and its derivatives,^21,22^ and bis(trifluoromethyl)pyrazoles compounds, initially named BTP (BTP1 (**1**), BTP2 (**2**), Figure 1),^23^ followed by Pyr’s (Pyr3 (**3**), Pyr6 (**4**), Pyr10 (**5**), Figure 1).^24,25^ Among others, GSK-7975A (**6**, Figure 1),^26^ RO2959 (**7**, Figure 1),^27^ and Synta66 (**8**, Figure 1)^11,12^ have been extensively used as chemical probes. More recently, two compounds have entered clinical development, CM4620 (**9**, Figure 1) for acute pancreatitis (phase II)^28^ and PRCL-02 for psoriasis (phase II),^29^ whose structure has not been disclosed. These are not the only SOCE inhibitors that are in human use. Indeed, a virtual screening performed on FDA-approved drugs recently unveiled that two approved drugs are also SOCE inhibitors with relevant activity at therapeutic doses,^30^ the prodrug leflunomide (**10**, Figure 1)^31,32^ and its active form teriflunomide (**11**, Figure 1).^33,34^ The two compounds are approved for the treatment of rheumatoid arthritis and multiple sclerosis, respectively. Teriflunomide is a dihydroorotate dehydrogenase (DHODH) inhibitor, while leflunomide is devoid of *in vitro* activity on this enzyme.

**Figure 1:**
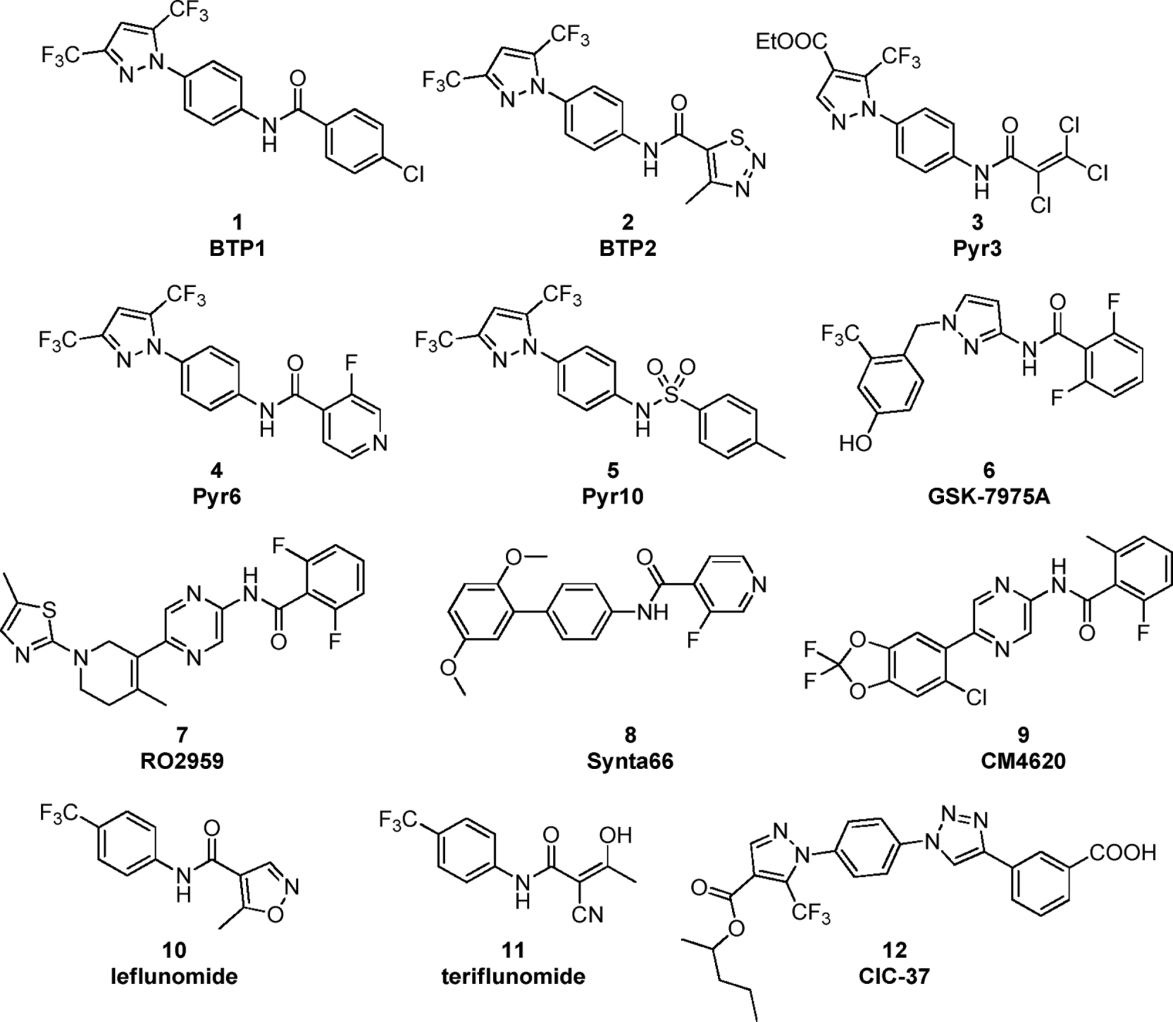
Structures of SOCE inhibitors reported in the literature.

Our group of research has contributed to the development of druglike SOCE modulators by reporting pyrtriazoles, among which the most promising candidate is CIC-37 (**12**, Figure 1).^35^

In contrast to other SOCE modulators, CIC-37 is a promising molecule as it displays selectivity over other calcium channels, such as TRPV1, TRPM8, and voltage-operated calcium channels.^35^ However, the molecule is affected by poor metabolic stability, and this is attributable to the ester group, which is shared by pyrtriazoles and some of the Pyr’s (**3**, Figure 1). Yet, this soft spot represents a fundamental portion in the pharmacophore of pyrtriazoles as its removal leads to a drop in the inhibitory activity, as previously described by our group.^35^ Therefore, in the present contribution, starting from the structure of CIC-37 (**12**, Figure 1), we replaced the ester moiety with a hydrolytically stable isostere, a 1,2,4-oxadiazole.^36,37^ Moreover, in the design of this novel class, we substituted the 1,4-disubstituted 1,2,3-triazole ring of compound **12** with an amide group (Figure 2). Indeed, the development of 1,2,3-triazoles is hampered by several issues, including poor aqueous solubility, limitations in the scale-up process due to the explosive nature of azides and safety concerns associated with copper catalysts.^38^

**Figure 2:**
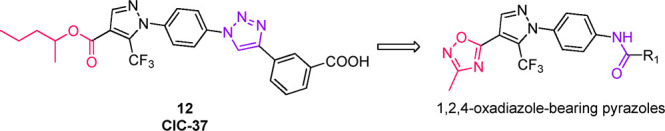
Design of 1,2,4-oxadiazole-bearing pyrazoles.

The class of oxadiazole-bearing pyrazoles was prepared from amine **17** (Scheme 1). The synthetic route consists of three steps: after a condensation between **13** and **14** and a reduction of the aromatic nitro group, intermediate **16** was obtained. Then, the ethyl ester reacted with *N*-hydroxyacetamidine to afford amine **17**.

**Scheme 1:**
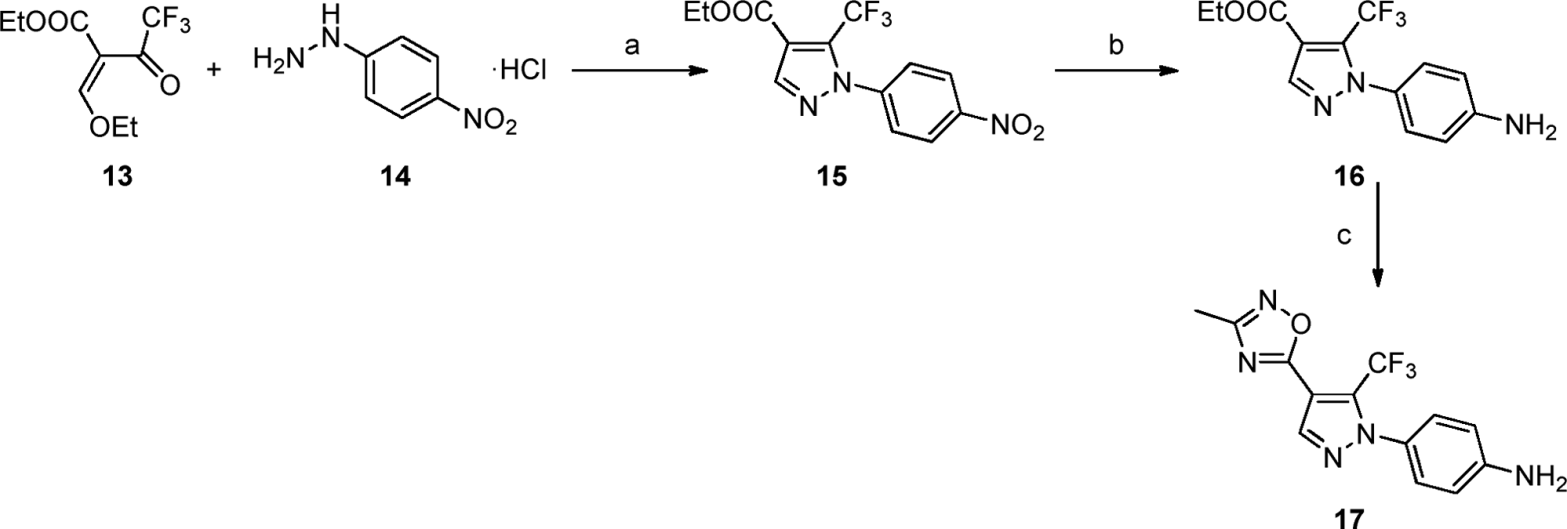
Preparation of Amine **17** Reagents and conditions: (a) DMF, reflux, 2 h, 96%. (b) H_2_, 5% Pd/C, EtOAc, rt, 2 h, 94%. (c) NaH 60%, *N*-hydroxyacetamidine, dry THF, 0 °C to rt, 4 h, 76%.

Starting from this amine, a structure–activity relationship (SAR) study was undertaken. To this aim, eight coupling reactions were performed (**19**–**23**, **32**–**33**, and **36**, Scheme 2) based on the prototype substructures displayed by reported SOCE inhibitors, including BTP1 (**1**, Figure 1), BTP2 (**2**, Figure 1), Pyr10 (**5**, Figure 1), GSK-7975A (**6**, Figure 1), Synta66 (**8**, Figure 1), CM4620 (**9**, Figure 1), leflunomide (**10**), and CIC-37 (**12**, Figure 1). Compound **42**, whose substructure mimics Pyr10 (**5**, Figure 1), was synthesized according to Scheme 3, from amine **17** and tosyl chloride (TsCl). Moreover, 15 additional coupling reactions were performed (**24**–**31**, **34**–**35**, and **37**–**41**, Scheme 2) exploiting different carboxylic acids and further expanding the SAR study.

**Scheme 2:**
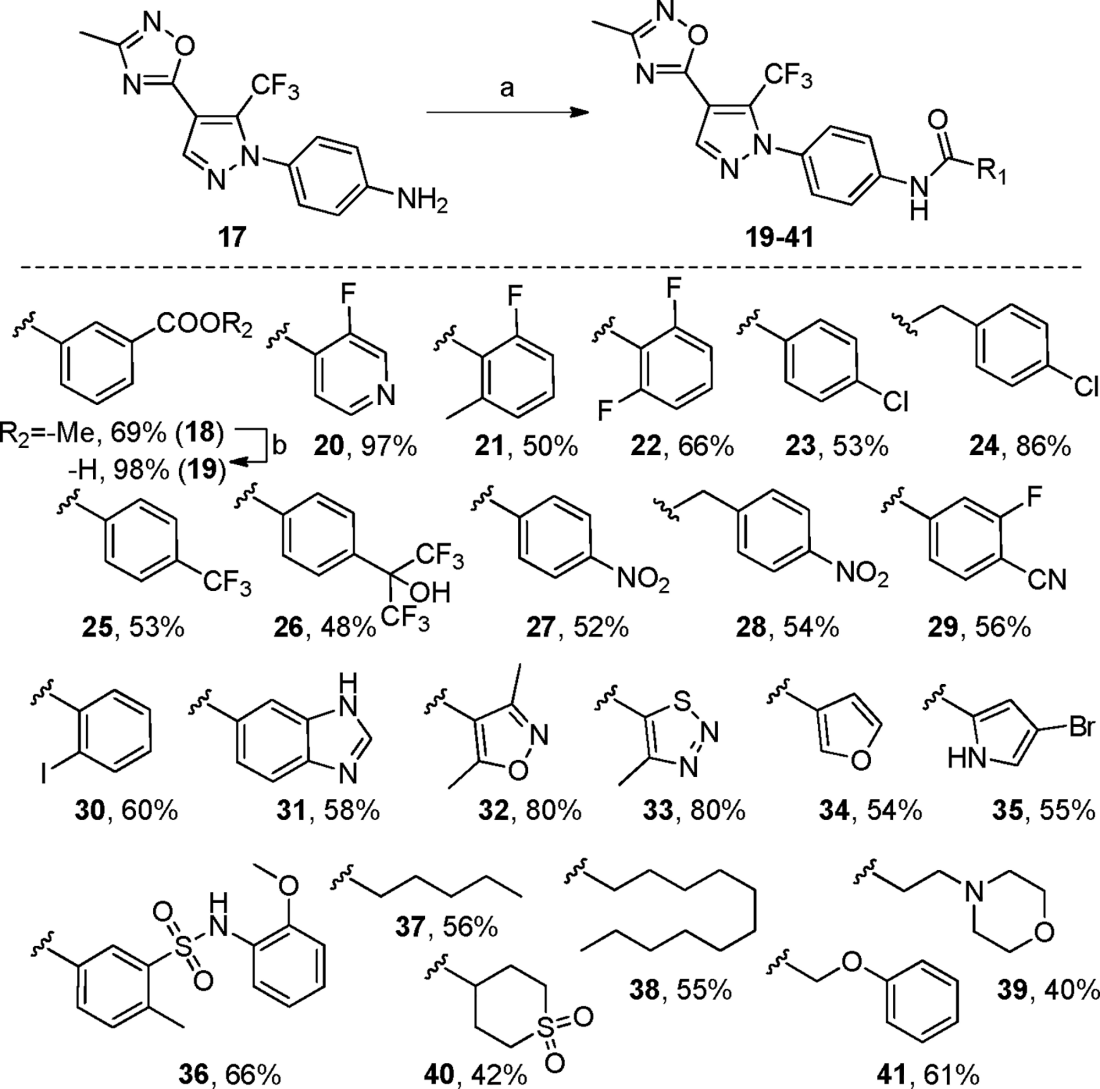
Preparation of Oxadiazole-Bearing Pyrazoles **19**–**41** Reagents and conditions: (a) PyBOP, DIPEA, dry CH_2_Cl_2_, rt, 16–42 h, 40–97%. (b) NaOH, H_2_O, THF, rt, 5 h, 98%.

**Scheme 3:**
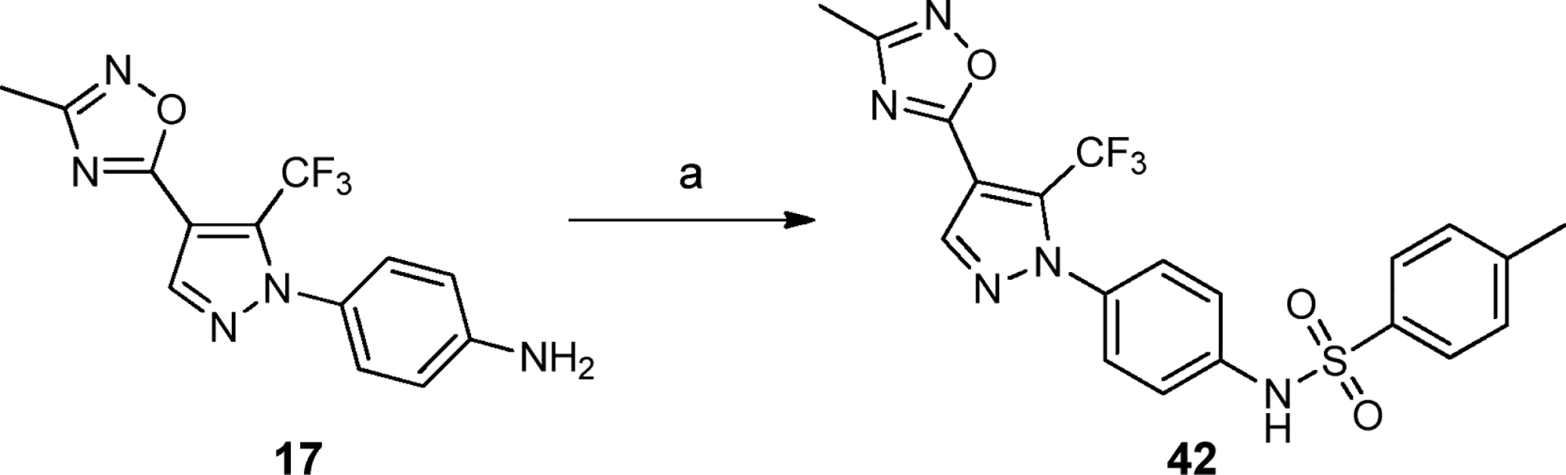
Synthesis of compound **42** Reagents and conditions: (a) TsCl, pyridine, dry CH_2_Cl_2_, 0 °C to rt, 5 h, 45%.

With this approach, 24 oxadiazole-bearing pyrazoles were synthesized and tested in human embryonic kidney (HEK) cells for SOCE inhibition. To this aim, intracellular Ca^2+^ stores of HEK cells were emptied with *t*-butylhydroquinone (*t*BhQ, 50 μM) in the presence of the compounds at 10 μM concentration. After 600 s, Ca^2+^ was added and intracellular levels were measured in fluorescence microscopy with the calcium dye Fura-2. As shown in Table 1, 8 compounds out of 24 were able to reduce calcium entry by more than 50% compared to control. In particular, the substructures that provided the highest inhibitory activity were those derived from GSK-7975A (**22**, % of SOCE residual activity: 38.6%), BTP1 (**23**, 40.8%), leflunomide (**32**, 33.2%), Pyr2 (**33**, 46.5%), and Pyr10 (**42**, 47.6%). Also, compounds **27** (40.8%) and **29** (49.6%), displaying an electron-withdrawing group in para position on the aromatic ring, or **31** (47.4%) in which the aromatic ring is fused with an imidazole, afforded a good inhibitory activity. For these compounds, the cell viability was therefore evaluated. An MTT assay was performed and the compounds that affected cell viability by more than 25% at 10 μM were discarded (**23**, **31**, and **33**). Notably, the remaining five compounds (**22**, **27**, **29**, **32**, and **42**) were not cytotoxic under these conditions. According to both activity and cytotoxicity, for the selected five compounds, the representative traces are depicted in Figure 3.

**Figure 3:**
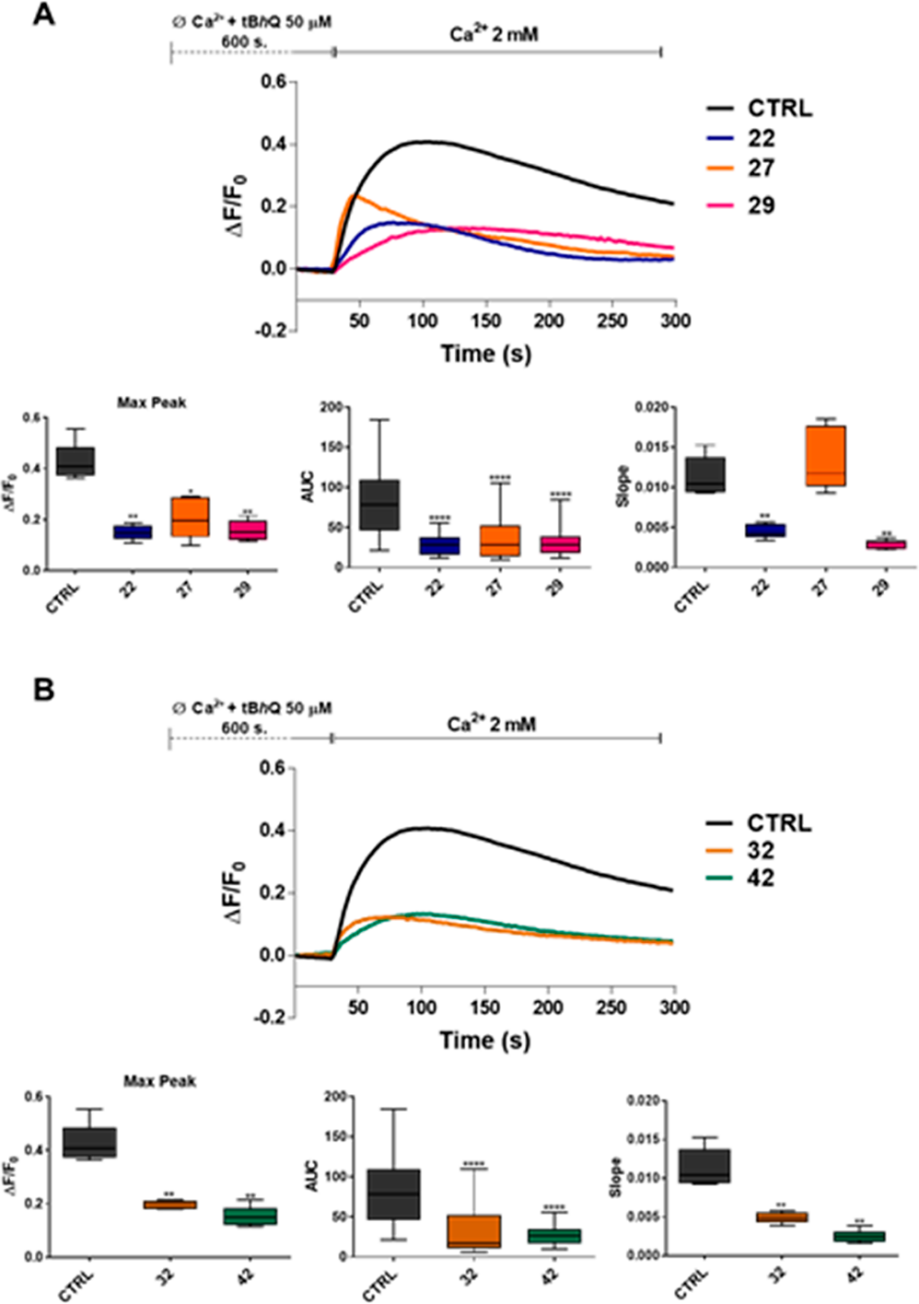
Effect of compounds **22**, **27**, **29**, **32**, and **42** on SOCE. (A) Traces of SOCE in the presence or absence of compounds **22**, **27**, and **29** (10 μM). Traces are the average of 200 cells from three independent experiments (top panel). Evaluation of peak amplitude, area under the curve (AUC), and slope of the Ca^2+^-rise in the absence or presence of **22**, **27**, and **29**. The graph shows the median and interquartile range (IQR) of the peak amplitude, AUC, and slope of the Ca^2+^-rise. Mann–Whitney U test of compound vs control (** *p* ≤ 0.0043; **** *p* ≤ 0.0001; bottom panel). (B) Traces of SOCE in the presence or absence of compounds **32** and **42** (10 μM). Traces are the average of 200 cells from three independent experiments (top panel). Evaluation of peak amplitude, AUC, and slope of the Ca^2+^-rise in the absence or presence of **32** and **42**. The graph shows the median and IQR of the peak amplitude, AUC, and slope of the Ca^2+^-rise. Mann–Whitney U test of compound vs control (**p ≤ 0.0069; ****p ≤ 0.0001; bottom panel).

**Table 1:** Oxadiazole-Bearing Pyrazoles and Their Activity on SOCE, IC_50_ Values and Cytotoxicity in HEK Cells^a^

compd, yield (%)	% SOCE residual activity (10 μM)	% viability (10 μM)	IC_50_ (μM)	MLS9^b^/MLM^c^ stability (residual substrate)
Pyr3 (**3**)	8.9 ± 0.6	28.6 ± 0.5	0.5 ± 0.1	43%^b^
Synta66 (**8**)	9.2 ± 1.7	75.8 ± 8.0	0.2 ± 0.3	15%^c^
CIC-37 (**12**)	17.5 ± 1.6	93.6 ± 4.6	4.4 ± 1.2	74%^b^
**19**, 69%	77.2 ± 3.7			
**20**, 97%	53.6 ± 0.9			
**21**, 50%	56.7 ± 1.3			
**22**, 66%	38.6 ± 4.3	95.6 ± 8.5	3.1 ± 1.4	>99%^b^
**23**, 53%	40.8 ± 3.6	72.9 ± 11.7		
**24**, 86%	121.1 ± 2.2			
**25**, 53%	110.0 ± 5.9			
**26**, 48%	109.0 ± 6.5			
**27**, 52%	40.8 ± 6.9	80.2 ± 7.4	5.5 ± 0.8	>99%^b^
**28**, 54%	107.9 ± 3.9			
**29**, 56%	49.6 ± 6.5	100.8 ± 6.7	9.7 ± 2.3	73%^b^
**30**, 60%	53.2 ± 3.9			
**31**, 58%	47.4 ± 1.0	56.5 ± 2.5		
**32**, 80%	33.2 ± 1.9	77.7 ± 4.4	9.6 ± 2.5	99%^b^
**33**, 80%	46.5 ± 2.8	59.1 ± 6.3		
**34**, 54%	61.1 ± 3.9			
**35**, 55%	69.8 ± 2.9			
**36**, 66%	59.9 ± 4.2			
**37**, 56%	146.2 ± 4.5	88. Five ±2.9		91%^b^
**38**, 55%	53.8 ± 4.4			
**39**, 40%	95.9 ± 6.2			
**40**, 42%	87.5 ± 3.8			
**41**, 61%	63.6 ± 3.9			
**42**, 45%	47.6 ± 4.2	79.4 ± 4.2	9.5 ± 1.7	76%^b^

a Biological data were derived for three independent experiments, and numbers represent mean ± standard error of mean (SEM).

b Residual substrates were determined in the MLS9 fraction after 1 h of incubation.

c Residual substrates were determined in mouse liver microsomes (MLM) as previously described.^39^

Moreover, for these selected compounds, IC_50_ values were calculated and are reported in Table 1. The most potent molecule was **22**, with an IC_50_ value of 3.1 μM.

Surprisingly, compound **37**, in which the side chain is represented by a linear aliphatic substructure, afforded an increase in calcium entry, with a percentage of SOCE activity of 146.2% compared to the control. More in detail, the compound, tested at the concentration of 10 μM, significantly increased the area under the curve (AUC) of calcium entry and the peak amplitude, without affecting the slope (Figure 4). We also investigated whether **37** required the triggered opening of the Orai channel to elicit its effect. To do this, we monitored intracellular Ca^2+^ in resting cells in the presence of extracellular Ca^2+^ and we observed that the compound did not elicit any significant calcium entry at 10 μM compared to control in the 300 s of observation, suggesting that **37** is a potentiator/enhancer of SOCE. The same effect was also evident at 3 (118.2%) and 1 μM (116.7%) but was not detected at concentrations above 10. In particular, at 30 and 100 μM, **37** acts as weak SOCE inhibitor, with a percentage of residual activity of about 70%.

**Figure 4:**
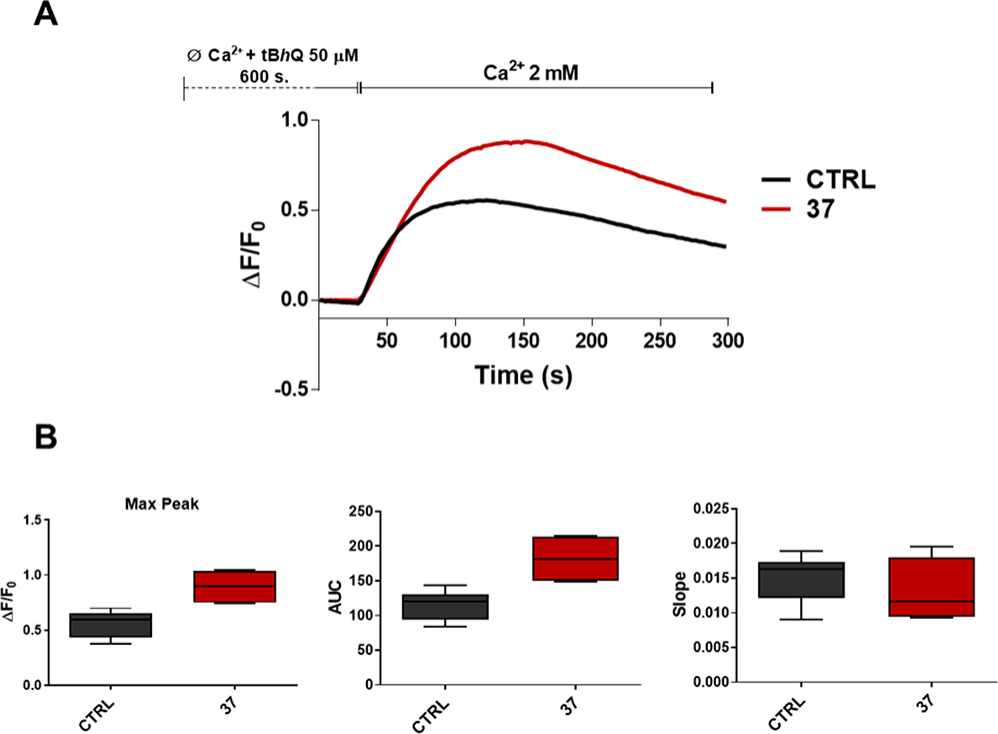
Effect of **37** on SOCE. (A) Traces of SOCE in the presence or absence of **37** at a concentration of 10 μM. Traces are the average of 200 cells from three independent experiments. (B) Evaluation of peak amplitude, AUC, and slope of the Ca^2+^-rise in the absence or presence of **37**. The graph shows the median and IQR of the peak amplitude, AUC, and slope of the Ca^2+^-rise. Mann–Whitney U test of compound vs control (**p ≤ 0.002).

The identification of a SOCE enhancer among a class of inhibitors has been reported in three previously published classes of modulators, with two of them described by us and represented by pyrtriazoles (AL-2T (**43**), NM-3G (**44**), Figure 5),^35^ and biphenyl triazoles (compound **45**, Figure 5)^39^ and one reported in the literature (IA65 (**46**), Figure 5).^40^ A similar behavior is also shared by 2-APB (**47**), a well-known inhibitor of IP3 receptors and TRP channels. The compound is a SOCE modifier in Orai1- and Orai3-expressing cells, acting as SOCE enhancer at low concentrations, while high concentrations induce a transient increase followed by complete inhibition.^41^ Although a precise rationale can not be extrapolated from the scaffolds of the enhancers, in our experience it often occurs that a minimal structural modification in a class of compounds designed to negatively modulate SOCE is capable of turning an inhibitor into a molecule able to increase calcium entry.

**Figure 5:**
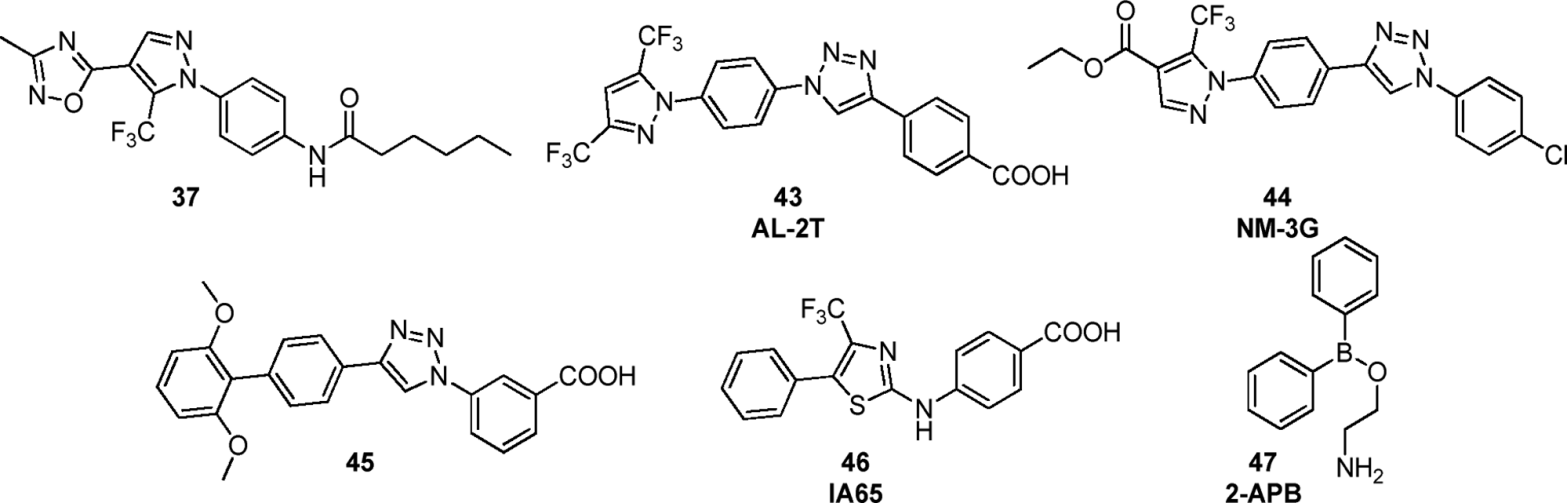
Structures of positive modulators of SOCE reported in the literature.

The five selected inhibitors (**22**, **27**, **29**, **32**, and **42**) and the identified SOCE enhancer (**37**) were then evaluated for their *in vitro* hepatic metabolic stability. To this aim, the candidates were incubated in MLS9 fraction supplied with NADPH and the residual substrate was measured after 1 h. Under these conditions, the oxadiazole-bearing pyrazoles resulted overall metabolically stable, with only two compounds affording a residual substrate lower than 80% and the remaining molecules higher than 90% after incubation (Table 1 and Supporting Information). In contrast, Pyr3 and CIC-37, which suffered from hydrolysis of the ester function, provided a residual substrate of 43 and 74%, respectively (Table 1).^35^ Similarly, the residual substrate of Synta66 in MLM is of 15%, due to an important hydrolysis of the amide group, absent in our class (Table 1).^39^

We have previously reported CIC-37 that had as its main advantage over other modulators the absence of cytotoxicity but had metabolic stability as its Achille’s heel. The most potent compound in the present contribution, **22**, is similarly not cytotoxic but shows a major improvement in *in vitro* metabolic stability (Table 1). Compared to the reference compounds of the literature (Pyr3 and Synta66), therefore, our newest compound does not affect cellular viability, displays a similar efficacy, but shows a significantly improved metabolic stability (Table 1).

In our SAR study, a SOCE enhancer has been identified, adding this compound to the previously reported pool of positive modulators. Due to poor available crystallographic data, the binding partner of the reported SOCE modulators has not been conclusively ascertained, but the presence of activators in our series strongly suggests that it is the Orai channel. The interaction of SOCE modulators with the ion channel is also supported by a recently published article in which, through computational approaches, a docking pose of Synta66 in the Orai channel has been proposed.^42^ Moreover, azopyrazole-derived SOCE inhibitors have been recently reported as the first photoswitchable SOCE modulators able to induce the activation of Orai using light, further supporting the hypothesis that our compounds interact directly with this ion channel.^43^

On one hand, SOCE inhibitors are currently being investigated for their potential therapeutic applications and have progressed in clinical trials; on the other hand, activators are still in their infancy, representing both chemical probes to better elucidate SOCE biology and hit compounds for the development of agents able to boost the immune system in loss-of-function mutations associated with severe combined immunodeficiency (SCID)-like disorders.^44,45^

## Supporting Information Available

Synthesis and characterization of compounds; in vitro metabolism and purity of lead compounds; biology methods (cell cultures, MTT assay, and calcium imaging) (PDF)

## Abbreviations

AUC: area under the curve
DHODH: dihydroorotate dehydrogenase
DIPEA: *N*,*N*-diisopropylethylamine
DMF: *N*,*N*-dimethylformamide
ER: endoplasmic reticulum
EtOAc: ethyl acetate
HEK: human embryonic kidney
IQR: interquartile range
MLM: mouse liver microsomes
MLS9: mouse liver S9
PyBOP: benzotriazol-1-yloxytripyrrolidinophosphonium hexafluorophosphate
SAR: structure–activity relationship
SEM: standard error of mean
SERCA: sarco-endoplasmic reticulum calcium ATPase
SCID: severe combined immunodeficiency
SOCE: Store-Operated Calcium Entry
THF: tetrahydrofuran
TsCl: tosyl chloride
tBhQ: *t*-butylhydroquinone
