# Supplementary material for: 1,2,4-Oxadiazole-Bearing Pyrazoles as Metabolically Stable Modulators of Store-Operated Calcium Entry
Source: ACS Med Chem Lett. 2021 Mar 10;12(4):640–6. doi: 10.1021/acsmedchemlett.1c00034 (PMC8040252; doi:10.1021/acsmedchemlett.1c00034)
Supplement: Supplementary file 1 — ml1c00034_si_001.pdf [file ml1c00034_si_001.pdf]

SUPPORTING INFORMATION

1,2,4-Oxadiazole-bearing pyrazoles as metabolically stable modulators of Store-Operated Calcium Entry

Silvio Aprile,[a] Beatrice Riva,[a,b] Irene Preet Bhela,[a] Celia Cordero-Sanchez,[a] Giulia Avino,[c] Armando A. Genazzani,[a] Marta Serafini,*[a] and Tracey Pirali[a,b]

[a] Department of Pharmaceutical Sciences; Università del Piemonte Orientale; Largo Donegani 2, 28100 Novara, Italy; [b] ChemICare S.r.l.; Enne3; Corso Trieste 15/A, 28100 Novara, Italy; [c] Department of Pharmaceutical Sciences; Università degli Studi di Trieste; Via Giorgieri 1, 34127 Trieste, Italy.

* marta.serafini@uniupo.it

Table of contents

p. S2 Chemistry

p. S19 *In vitro* metabolism and purity of lead compounds

p. S21 Biology

p. S23 References

Chemistry

General Experimental Methods. Commercially available reagents and solvents were used as purchased without further purification. When needed, solvents were distilled and stored on molecular sieves. Column chromatography was performed on silica gel. Thin layer chromatography (TLC) was carried out on 5 cm × 20 cm plates with a layer thickness of 0.25 mm. When necessary, TLC plates were visualized with aqueous $KMnO_4$ or with aqueous Pancaldi solution. Melting points were determined in open glass capillary with a Stuart scientific SMP3 apparatus. All the target compounds were checked by IR (FT-IR Bruker Alpha II), ^{1}H-NMR (Bruker Avance Neo 400 MHz; Jeol ECP 300 MHz), ^{13}C-NMR (Bruker Avance Neo 400 MHz), and mass spectrometry (Thermo Scientific Q-Exactive Plus) equipped with an HESI source. Chemical shifts are reported in parts per million (ppm). For all the target compounds, purity was checked by HPLC-UV (Shimadzu LC-10 system equipped with a SPD-M10Avp photodiode array detector) and their purity was ≥95%. Carboxylic acids are commercially available, with the only exception of 3-(*N*-(2-methoxyphenyl)sulfamoyl)-4-methylbenzoic acid that was synthesized as reported below. The reference compound Pyr3 was purchased from Sigma Aldrich (purity ≥98%, HPLC), while CIC-37 was synthesized as previously described[1] and its purity was ≥95%, assessed as previously described.[1]

4-(4-(3-Methyl-1,2,4-oxadiazol-5-yl)-5-(trifluoromethyl)-1*H*-pyrazol-1-yl)aniline, (17).

A solution of NaH (361 mg, 60% in mineral oil, 9.03 mmol) was added portionwise to a round bottom flask containing dry THF (120 mL) and *N*-hydroxyacetamidine (669 mg, 9.03 mmol) was added at 0 °C and under nitrogen. The mixture was left to reach rt and heated at 50 °C for 1 h. Then, ethyl 1-(4-aminophenyl)-5-(trifluoromethyl)-1*H*-pyrazole-4-carboxylate **17** (2.70 g, 9.03 mmol) was added and the reaction was heated at 50 °C for 3 h, diluted with EtOAc and washed with water (2x). The organic layer was dried over sodium sulfate and evaporated, yielded compound **18** as a yellow solid (2.12 g, 6.86 mmol, 76%). ^{1}H-NMR (400 MHz; $CDCl_3$): δ 8.19 (s, 1H), 7.18 (d, J = 8.6 Hz, 2H), 6.66 (d, J = 8.6 Hz, 2H), 4.07 (br s, 2H), 2.43 (s, 3H). HRMS (ESI) m/z $(M+H)^+$ calcd for $C_{13}H_{11}F_3N_5O$ 310.0916, found 310.0921.

General procedure for the synthesis of compounds 19-41.

4-(4-(3-Methyl-1,2,4-oxadiazol-5-yl)-5-(trifluoromethyl)-1*H*-pyrazol-1-yl)aniline **17** (100 mg, 0.32 mmol) was solubilized in dry CH_2Cl_2 (2.5 mL) and DIPEA (109 µL 0.64 mmol), PyBOP (166 mg, 0.32 mmol) and the carboxylic acid (0.35 mmol, 1.1 eq) were added in order and under nitrogen. After 16 h, DIPEA (0.6 eq), PyBOP (0.6 eq) and the carboxylic acid (0.3 eq) were added if necessary and the mixture was stirred for additional 16 h. In case that the reaction was not concluded, DIPEA (0.3 eq), PyBOP (0.3 eq) and the carboxylic acid (0.3 eq) were re-added and after additional 16 h, the reaction was worked-up. The mixture was diluted with CH_2Cl_2 and washed with water (2x). The organic layer was dried over sodium sulfate and evaporated. The crude product was purified by column chromatography.

3-((4-(4-(3-Methyl-1,2,4-oxadiazol-5-yl)-5-(trifluoromethyl)-1*H*-pyrazol-1-yl)phenyl)carbamoyl)benzoic acid, (19).

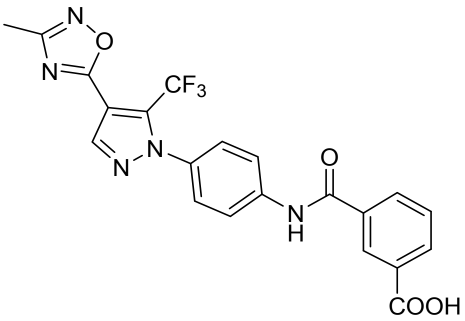

Methyl 3-((4-(4-(3-methyl-1,2,4-oxadiazol-5-yl)-5-(trifluoromethyl)-1*H*-pyrazol-1-yl)phenyl)carbamoyl)benzoate was prepared following the general procedure starting from amine **17** (100 mg, 0.32 mmol) and 3-(methoxycarbonyl)benzoic acid (63 mg, 0.35 mmol). Purification by column chromatography using PE/EtOAc 6:4 afforded compound **18** as a white solid (104 mg, 0.22 mmol, 69%). The intermediate was then solubilized in THF (2 mL) and water (2 mL) and NaOH (17.6 mg, 0.44 mmol) were added. After 5 h, the mixture was diluted with water and HCl 3N was added until pH 4. The aqueous layer was extracted with EtOAc (2x). The collected organic layers were dried over sodium sulfate and evaporated. The crude material was purified by column chromatography using PE/EtOAc 3:7 as eluent affording compound **19** (99 mg, 0.22 mmol, 98%) as

an amorphous white solid. ^{1}H-NMR (400 MHz; CD_3OD): δ 8.65 (s, 1H), 8.38 (s, 1H), 8.26 (d, *J* = 7.8 Hz, 1H), 8.21 (d, *J* = 7.8 Hz, 1H), 8.02 (d, *J* = 8.8 Hz, 2H), 7.67 (t, *J* = 7.8 Hz, 1H), 7.56 (d, *J* = 8.8 Hz, 2H), 2.48 (s, 3H). ^{13}C-NMR (101 MHz; CD_3OD): δ 168.7, 167.8, 167.5, 166.7, 141.0, 140.6, 135.1, 134.6, 132.6, 131.6, 131.2 (q, *J* = 45.4 Hz), 128.6, 128.6, 126.7, 126.4, 120.8, 119.2 (q, *J* = 271.6 Hz), 108.9, 9.9. IR (neat): $\tilde{\nu}$ = 3270, 2922, 2853, 1700, 1650, 1519, 1298, 1141, 947, 808 cm^{-1}. HRMS (ESI) *m/z* $(M+H)^+$ calcd for $C_{21}H_{15}F_3N_5O_4$ 458.1071, found 458.1071.

Characterization of compounds 20-41.

3-Fluoro-*N*-(4-(4-(3-methyl-1,2,4-oxadiazol-5-yl)-5-(trifluoromethyl)-1*H*-pyrazol-1-yl)phenyl)isonicotinamide, (20).

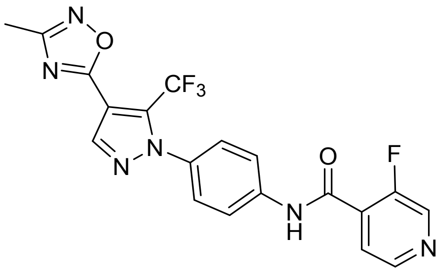

Compound **20** was synthesized following the general procedure starting from compound **17** (100 mg, 0.32 mmol) and 3-fluoroisonicotinic acid (49.3 mg, 0.35 mmol). White solid (134 mg, 0.31 mmol, 97%); Chromatography: PE/EtOAc 5:5; mp: 185-186 °C, dec. ^{1}H-NMR (400 MHz; $(CD_3)_2CO$): δ 8.70-8.63 (m, 2H), 8.39 (s, 1H), 8.08 (d, *J* = 7.7 Hz, 2H), 7.81 (s, 1H), 7.67 (d, *J* = 7.7 Hz, 2H), 7.43 (br s, 1H), 2.46 (s, 3H). ^{13}C-NMR (101 MHz; $(CD_3)_2CO$): δ 168.6, 167.9, 161.0, 155.7 (d, *J* = 253.3 Hz), 146.4 (d, *J* = 5.2 Hz), 141.1, 140.2, 139.1 (d, *J* = 24.7 Hz), 138.4 (d, *J* = 23.5 Hz), 135.0, 130.6 (q, *J* = 32.4 Hz), 126.9, 123.4, 120.3, 119.3 (q, *J* = 271.9 Hz), 109.2, 10.6. IR (neat): $\tilde{\nu}$ = 3487, 2922, 2852, 1630, 1516, 1422, 1296, 1146, 844, 508 cm^{-1}. HRMS (ESI) *m/z* $(M+H)^+$ calcd for $C_{19}H_{13}F_4N_6O_2$ 433.1031, found 433.1027.

2-Fluoro-6-methyl-*N*-(4-(4-(3-methyl-1,2,4-oxadiazol-5-yl)-5-(trifluoromethyl)-1*H*-pyrazol-1-yl)phenyl)benzamide, (21).

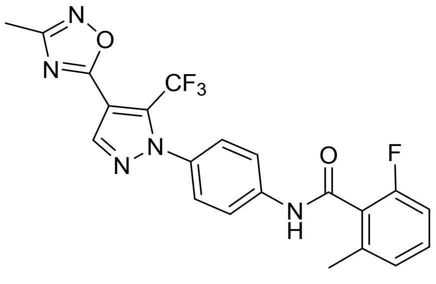

Compound **21** was synthesized following the general procedure starting from compound **17** (100 mg, 0.32 mmol) and 2-fluoro-6-methylbenzoic acid (53.9 mg, 0.35 mmol). White solid (71 mg, 0.16 mmol, 50%); Chromatography: PE/EtOAc 9:1; mp: 218-219 °C ^{1}H-NMR (400 MHz; $CDCl_3$): δ 8.26 (s, 1H), 7.84 (d, *J* = 8.4 Hz, 2H), 7.50 (d, *J* = 8.4 Hz, 2H), 7.37-7-32 (m, 1H), 7.10 (d, *J* = 7.6 Hz, 1H), 7.04-6.99 (m, 1H), 2.50-2.48 (m, 6H). ^{13}C-NMR (101 MHz; $CDCl_3$): δ 168.6, 167.8, 163.4, 159.3 (d, *J* = 247.0 Hz), 141.3, 139.3, 139.2 (d, *J* = 2.3 Hz), 134.9, 131.4 (q, *J* = 40.7 Hz), 131.3 (d, *J* = 9.0 Hz), 126.7, 126.6, 124.1 (d, *J* = 16.7 Hz), 120.1, 118.9 (q, *J* = 272.8 Hz), 113.3 (d, *J* = 22.2 Hz), 109.3, 19.5, 11.6. IR (neat): $\tilde{\nu}$ = 3246, 2918, 2850, 1656, 1512, 1298, 1136, 944, 792, 588 cm^{-1}. HRMS (ESI) *m/z* $(M+H)^+$ calcd for $C_{21}H_{16}F_4N_5O_2$ 446.1235, found 446.1231.

2,6-Difluoro-*N*-(4-(4-(3-methyl-1,2,4-oxadiazol-5-yl)-5-(trifluoromethyl)-1*H*-pyrazol-1-yl)phenyl)benzamide, (22).

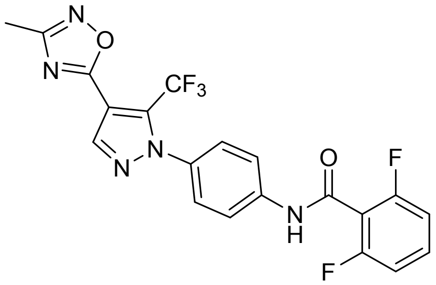

Compound **22** was synthesized following the general procedure starting from compound **17** (100 mg, 0.32 mmol) and 2,6-difluorobenzoic acid (55.3 mg, 0.35 mmol). White solid (95 mg, 0.21 mmol, 66%); Chromatography: PE/EtOAc 7:3; mp: 180-181 °C. ^{1}H-NMR 400 MHz; $CDCl_3$): δ 8.25 (s, 1H), 8.16 (br s, 1H), 7.83 (d, *J* = 7.4 Hz, 2H), 7.48-7.42 (m, 3H), 7.01-6.99 (m, 2H), 2.51 (s, 3H). ^{13}C-NMR (101 MHz; $CDCl_3$): δ 168.6, 167.8, 160.0 (dd, *Js* = 254.4, 6.5 Hz), 158.6, 141.3, 139.1, 135.1, 132.5 (t, *J* = 10.4 Hz), 131.4 (q, *J* = 41.2 Hz), 126.7, 120.4, 119.4 (q, *J* = 272.3 Hz), 113.9 (t, *J* =

18.9 Hz), 112.3 (d, J = 25.5 Hz), 109.3, 11.6. IR (neat): $\tilde{\nu}$ = 3282, 2922, 2852, 1656, 1513, 1467, 1145, 1007, 794, 658 cm^{-1}. HRMS (ESI) *m/z* $(M+H)^+$ calcd for $C_{20}H_{13}F_5N_5O_2$ 450.0984, found 450.0981.

4-Chloro-*N*-(4-(4-(3-methyl-1,2,4-oxadiazol-5-yl)-5-(trifluoromethyl)-1*H*-pyrazol-1-yl)phenyl)benzamide, (23).

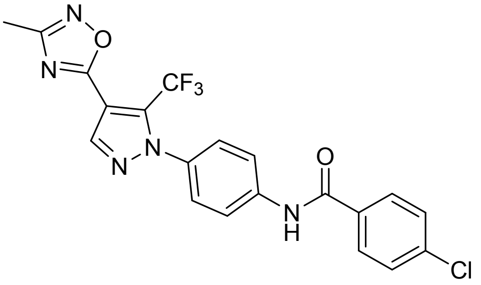

Compound **23** was synthesized following the general procedure starting from compound **17** (100 mg, 0.32 mmol) and 4-chlorobenzoic acid (54.8 mg, 0.35 mmol). White solid (76 mg, 0.17 mmol, 53%); Chromatography: PE/EtOAc 7:3; mp: 208-209 °C, dec. ^{1}H-NMR (400 MHz; $CDCl_3$): δ 8.28 (s, 1H), 8.07 (br s, 1H), 7.87-7.84 (m, 4H), 7.52-7.50 (m, 4H), 2.52 (s, 3H). ^{13}C-NMR (101 MHz; $CDCl_3$): δ 168.5, 167.9, 164.9, 141.3, 139.5, 138.9, 134.8, 132.8, 131.4 (q, *J* = 40.4 Hz), 129.2, 128.5, 126.7, 120.4, 119.0 (q, *J* = 273.7 Hz), 109.3, 11.6. IR (neat): $\tilde{\nu}$ = 3273, 2923, 2852, 1650, 1518, 1300, 1130, 840, 756, 535 cm^{-1}. HRMS (ESI) *m/z* $(M+H)^+$ calcd for $C_{20}H_{14}ClF_3N_5O_2$ 448.0783, found 448.0782.

2-(4-Chlorophenyl)-*N*-(4-(4-(3-methyl-1,2,4-oxadiazol-5-yl)-5-(trifluoromethyl)-1*H*-pyrazol-1-yl)phenyl)acetamide, (24).

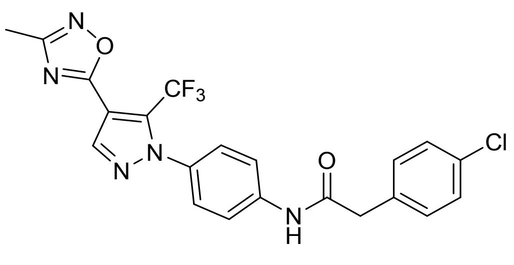

Compound **24** was synthesized following the general procedure starting from compound **17** (100 mg, 0.32 mmol) and 2-(4-chlorophenyl)acetic acid (59.7 mg, 0.35 mmol). White solid (127 mg, 0.28 mmol, 86%); Cromatography: PE/EtOAc 6:4; mp: 169-171 °C, dec. ^{1}H-NMR (400 MHz; $(CD_3)_2CO$):

δ 9.73 (br s, 1H), 8.38 (s, 1H), 7.91 (d, J = 8.0 Hz, 2H), 7.56 (d, J = 8.0 Hz, 2H), 7.44 (d, J = 8.0 Hz, 2H), 7.38 (d, J = 8.0 Hz, 2H), 3.80 (s, 2H), 2.45 (s, 3H). ^{13}C-NMR (101 MHz; $(CD_3)_2CO$): δ 169.0, 168.6, 167.8, 141.0, 134.5, 134.1, 132.2, 131.0, 130.8 (q, J = 43.8 Hz), 128.3, 126.7, 124.4, 119.4, 119.3 (q, J = 271.8 Hz), 109.4, 42.8, 10.6. IR (neat): $\tilde{\nu}$ = 3254, 2922, 2852, 1664, 1651, 1625, 1601, 1519, 977, 606 cm^{-1}. HRMS (ESI) *m/z* $(M+H)^+$ calcd for $C_{21}H_{16}ClF_3N_5O_2$ 462.0939, found 462.0935.

***N*-(4-(4-(3-Methyl-1,2,4-oxadiazol-5-yl)-5-(trifluoromethyl)-1*H*-pyrazol-1-yl)phenyl)-4-(trifluoromethyl)benzamide, (25).**

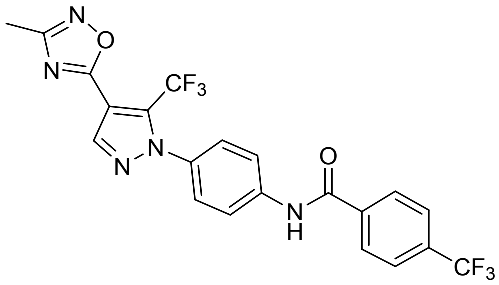

Compound **25** was synthesized following the general procedure starting from compound **17** (100 mg, 0.32 mmol) and 4-(trifluoromethyl)benzoic acid (66.5 mg, 0.35 mmol). White solid (81 mg, 0.17 mmol, 53%); Chromatography: PE/EtOAc 8:2; mp: 190-192 °C, dec. ^{1}H-NMR (400 MHz; $CDCl_3$, *: referred to the main rotamer): δ 8.26 (s, 1H), 8.05-8.03 (m, 3H)*, 7.89 (d, J = 8.7 Hz, 2H), 7.81 (d, J = 8.2 Hz, 2H)*, 7.54 (d, J = 8.7 Hz, 2H), 2.52 (s, 3H). ^{13}C-NMR (101 MHz; $(CD_3)_2CO$): δ 168.6, 167.5, 164.7, 141.1, 140.8, 134.6, 133.6 (q, J = 32.3 Hz), 132.6 (q, J = 32.5 Hz), 130.3, 128.4, 126.7, 125.5, 121.7 (q, J = 202.9 Hz), 120.4, 119.6 (q, J = 271.6 Hz), 109.2, 10.6. IR (neat): $\tilde{\nu}$ = 3248, 3098, 1655, 1628, 1518, 1287, 1139, 1104, 978, 699 cm^{-1}. HRMS (ESI) *m/z* $(M+H)^+$ calcd for $C_{21}H_{14}F_6N_5O_2$ 482.1046, found 482.1047.

4-(1,1,1,3,3,3-Hexafluoro-2-hydroxypropan-2-yl)-*N*-(4-(4-(3-methyl-1,2,4-oxadiazol-5-yl)-5-(trifluoromethyl)-1*H*-pyrazol-1-yl)phenyl)benzamide, (26).

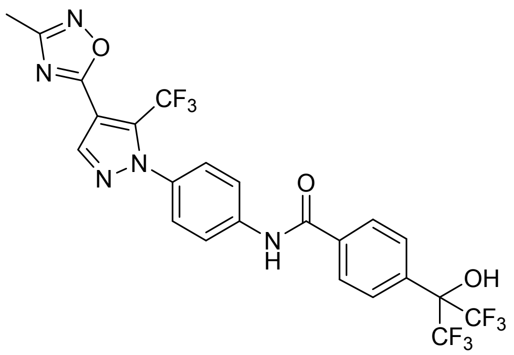

Compound **26** was synthesized following the general procedure starting from compound **17** (100 mg, 0.32 mmol) and 4-(1,1,1,3,3,3-hexafluoro-2-hydroxypropan-2-yl)benzoic acid (100.9 mg, 0.35 mmol). White solid (89 mg, 0.15 mmol, 48%); Chromatography: PE/EtOAc 8:2; mp: 180-182 °C, ^{1}H-NMR (400 MHz; $CDCl_3$): δ 8.29 (s, 1H), 8.01-7.99 (m, 3H), 7.92 (d, *J* = 8.2 Hz, 2H), 7.87 (d, *J* = 8.5 Hz, 2H), 7.54 (d, *J* = 8.5 Hz, 2H), 2.52 (s, 3H). ^{13}C-NMR (101 MHz; $(CD_3)_2CO$, *: referred to the main rotamer): δ 168.6*, 167.9*, 165.9, 165.2*, 165.1, 141.1*, 141.0, 140.9, 136.9*, 136.8, 134.5*, 134.1, 132.4, 130.8 (q, *J* = 40.2 Hz)*, 129.7*, 127.8*, 127.2*, 126.7*, 124.4, 123.5 (q, *J* = 37.7 Hz)*,123.0 (q, *J* = 288.5 Hz)*, 121.6, 120.3, 120.2, 119.3 (q, *J* = 271.9 Hz), 109.2, 10.5. IR (neat): $\tilde{\nu}$ = 3302, 1666, 1630, 1324, 1151, 1114, 975, 933, 718 cm^{-1}. HRMS (ESI) *m/z* $(M+H)^+$ calcd for $C_{23}H_{15}F_9N_5O_3$ 580.1026, found 580.1024.

***N*-(4-(4-(3-Methyl-1,2,4-oxadiazol-5-yl)-5-(trifluoromethyl)-1*H*-pyrazol-1-yl)phenyl)-4-nitrobenzamide, (27).**

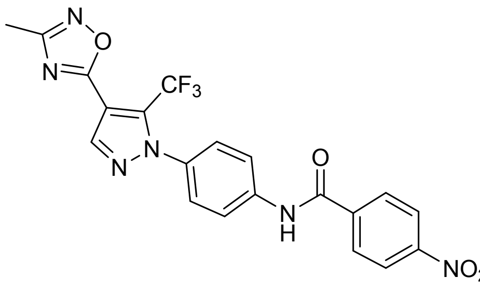

Compound **27** was synthesized following the general procedure starting from compound **17** (100 mg, 0.32 mmol) and 4-nitrobenzoic acid (58.5 mg, 0.35 mmol). White solid (76 mg, 0.17 mmol, 52%); Chromatography: PE/EtOAc 7:3; mp: 153-155 °C, dec. ^{1}H-NMR (400 MHz; $CDCl_3$): δ 8.40 (d, *J* = 8.5 Hz, 2H), 8.29 (s, 1H), 8.10 (d, *J* = 8.5 Hz, 2H), 8.07 (br s, 1H), 7.88 (d, *J* = 8.7 Hz, 2H), 7.55 (d,

J = 8.7 Hz, 2H), 2.52 (s, 3H). ^{13}C-NMR (101 MHz; $CDCl_3$): δ 168.5, 167.9, 163.8, 150.1, 141.4, 139.9, 138.9, 135.3, 131.4 (q, *J* = 40.4 Hz), 128.4, 126.9, 124.2, 120.5, 122.3 (q, *J* = 272.7 Hz), 109.5, 11.6. IR (neat): $\tilde{\nu}$ = 3236, 3092, 1655, 1632, 1598, 1518, 1140, 979, 834, 701 cm^{-1}. HRMS (ESI) *m/z* $(M+H)^+$ calcd for $C_{20}H_{14}F_3N_6O_4$ 459.1023, found 459.1021.

***N*-(4-(4-(3-Methyl-1,2,4-oxadiazol-5-yl)-5-(trifluoromethyl)-1*H*-pyrazol-1-yl)phenyl)-2-(4-nitrophenyl)acetamide, (28).**

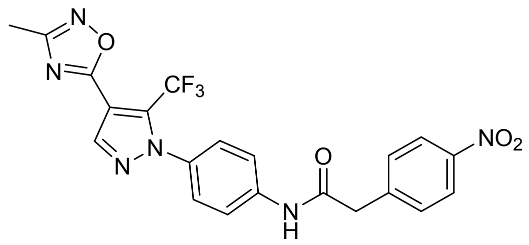

Compound **28** was synthesized following the general procedure starting from compound **17** (100 mg, 0.32 mmol) and 2-(4-nitrophenyl)acetic acid (63.4 mg, 0.35 mmol). White solid (81.6 mg, 0.17 mmol, 54%); Cromatography: PE/EtOAc 6:4; mp: 171-173 °C. ^{1}H-NMR (400 MHz; $CDCl_3$): δ 8.27-8.25 (m, 3H), 7.70 (d, *J* = 8.2 Hz, 2H), 7.60 (br s, 1H), 7.56 (d, *J* = 8.2 Hz, 2H), 7.45 (d, *J* = 8.0 Hz, 2H), 3.90 (s, 2H), 2.51 (s, 3H). ^{13}C-NMR (101 MHz; ; $(CD_3)_2CO$): δ 168.6, 168.2, 167.8, 147.1, 143.3, 141.0, 140.8, 134.2, 130.7 (q, *J* = 42.6 Hz), 130.6, 126.7, 123.3, 119.4, 119.2 (q, *J* = 271.5 Hz), 109.1, 43.1, 10.6. IR (neat): $\tilde{\nu}$ = 3255, 3115, 3080, 1662, 1629, 1599, 1535, 1518, 1133 cm^{-1}. HRMS (ESI) *m/z* $(M+H)^+$ calcd for $C_{21}H_{16}F_3N_6O_4$ 473.1180, found 473.1180.

4-Cyano-3-fluoro-*N*-(4-(4-(3-methyl-1,2,4-oxadiazol-5-yl)-5-(trifluoromethyl)-1*H*-pyrazol-1-yl)phenyl)benzamide, (29).

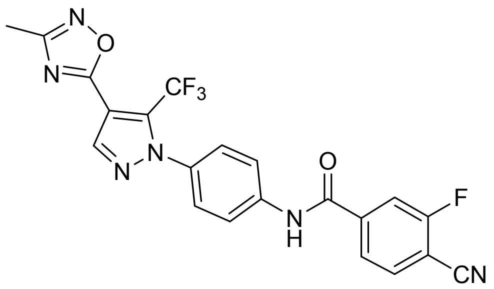

Compound **29** was synthesized following the general procedure starting from compound **17** (100 mg, 0.32 mmol) and 4-cyano-3-fluorobenzoic acid (57.8 mg, 0.35 mmol). White solid (82 mg, 0.18 mmol, 56%); Chromatography: PE/EtOAc 7:3; mp: 160-162 °C, dec. ^{1}H-NMR (400 MHz; $CDCl_3$): δ 8.30 (s, 1H), 7.87 (br s, 1H), 7.85-7.80 (m, 5H), 7.56 (d, *J* = 8.8 Hz, 2H), 2.52 (s, 3H). ^{13}C-NMR (101 MHz; DMSO-*d*$_6$): δ 168.6, 168.2, 163.8, 162.7 (d, *J* = 257.5 Hz), 141.9 (d, *J* = 7.1 Hz), 141.8, 140.7, 134.8, 134.5, 130.8 (q, *J* = 40.4 Hz), 127.2, 125.2 (d, *J* = 3.0 Hz), 121.1, 119.4 (q, *J* = 271.7 Hz), 116.2 (d, *J* = 22.2 Hz), 114.0, 108.9, 103.4 (d, *J* = 15.1 Hz), 11.6.

IR (neat): $\tilde{\nu}$ = 3364, 3080, 2921, 2239, 2197, 1680, 1632, 1139, 977, 757 cm^{-1}. HRMS (ESI) *m/z* $(M+H)^+$ calcd for $C_{21}H_{13}F_4N_6O_2$ 457.1031, found 457.1029.

2-Iodo-*N*-(4-(4-(3-methyl-1,2,4-oxadiazol-5-yl)-5-(trifluoromethyl)-1*H*-pyrazol-1-yl)phenyl)benzamide, (30).

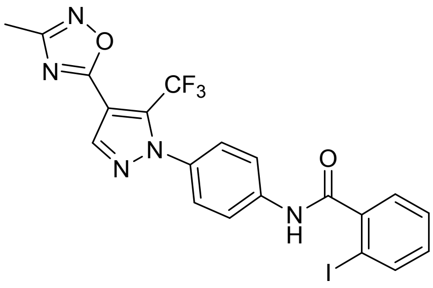

Compound **30** was synthesized following the general procedure starting from compound **17** (100 mg, 0.32 mmol) and 2-iodobenzoic acid (86.8 mg, 0.35 mmol). White solid (103 mg, 0.19 mmol, 60%); Chromatography: PE/EtOAc 7:3; mp: 201-203 °C, ^{1}H-NMR (400 MHz; $CDCl_3$): δ 8.27 (s, 1H), 7.96 (d, *J* = 7.9 Hz, 1H), 7.85 (d, *J* = 8.4 Hz, 2H), 7.70 (br s, 1H), 7.59 (d, *J* = 7.9 Hz, 1H), 7.53-7.47 (m, 3H), 7.21 (t, *J* = 7.9 Hz, 1H), 2.52 (s, 3H). ^{13}C-NMR (101 MHz; $CDCl_3$): δ 168.6, 167.8, 167.3, 141.7, 141.3, 140.2, 139.3, 134.9, 131.8, 131.4 (q, *J* = 40.8 Hz), 128.6, 128.4, 127.9, 126.7, 120.2, 119.0 (q, *J* = 272.6 Hz), 109.3, 92.3, 11.6. IR (neat): $\tilde{\nu}$ = 3310, 3097, 2921, 2851, 1687, 1631, 1153, 977, 756, 546 cm^{-1}. HRMS (ESI) *m/z* $(M+H)^+$ calcd for $C_{20}H_{14}F_3IN_5O_2$ 540.0139, found 540.0137.

***N*-(4-(4-(3-Methyl-1,2,4-oxadiazol-5-yl)-5-(trifluoromethyl)-1*H*-pyrazol-1-yl)phenyl)-1*H*-benzo[*d*]imidazole-6-carboxamide, (31).**

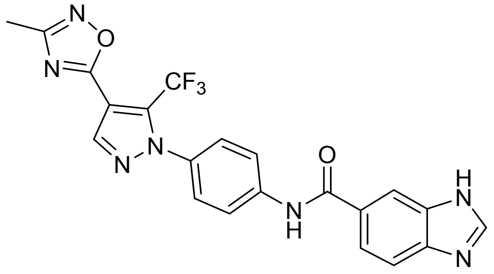

Compound **31** was synthesized following the general procedure starting from compound **17** (100 mg, 0.32 mmol) and 1*H*-benzo[*d*]imidazole-5-carboxylic acid (56.7 mg, 0.35 mmol). White solid (84 mg, 0.19 mmol, 58%); Chromatography: PE/EtOAc 1:9; mp: 235-236 °C. ^{1}H-NMR (400 MHz; CD_3OD): δ 8.38-8.33 (m, 2H), 8.03-8.01 (m, 3H), 7.96 (d, *J* = 8.5 Hz, 1H), 7.76 (dd, *Js* = 7.5, 1.6 Hz, 1H), 7.57 (d, *J* = 6.8 Hz, 2H), 2.48 (s, 3H). ^{13}C-NMR (101 MHz; DMSO-d_6): δ 168.6, 168.2, 166.9, 141.8 (2C), 133.8 (2C), 130.8 (q, *J* = 40.0 Hz), 128.5, 127.0 (2C), 123.1, 120.9, 119.5, 119.4 (q, *J* = 272.2 Hz), 117.6, 112.0, 108.9, 11.6. IR (neat): $\tilde{\nu}$ = 3637, 2921, 2851, 1626, 1519, 1293, 1140, 946, 830, 547 cm^{-1}. HRMS (ESI) *m/z* $(M+H)^+$ calcd for $C_{21}H_{15}F_3N_7O_2$ 454.1234, found 454.1231.

3,5-Dimethyl-*N*-(4-(4-(3-methyl-1,2,4-oxadiazol-5-yl)-5-(trifluoromethyl)-1*H*-pyrazol-1-yl)phenyl)isoxazole-4-carboxamide, (32).

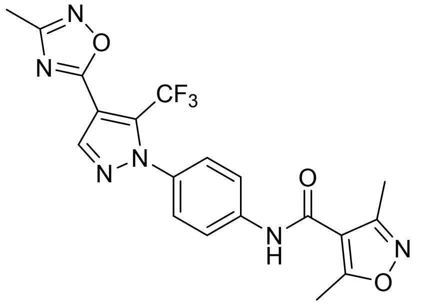

Compound **32** was synthesized following the general procedure starting from compound **17** (100 mg, 0.32 mmol) and 3,5-dimethylisoxazole-4-carboxylic acid (49.4 mg, 0.35 mmol). White solid (111 mg, 0.26 mmol, 80%); Chromatography: PE/EtOAc 8:2; mp: 177-178 °C, dec. ^{1}H-NMR (400 MHz; $CDCl_3$): δ 8.27 (s, 1H), 7.89 (br s, 1H), 7.78 (d, *J* = 6.6 Hz, 2H), 7.49 (d, *J* = 6.6 Hz, 2H), 2.68 (s, 3H), 2.50 (s, 3H), 2.48 (s, 3H). ^{13}C-NMR (101 MHz; $CDCl_3$): δ 172.0, 168.5, 167.8, 160.3, 157.6, 141.3, 139.1, 134.9, 131.4 (q, *J* = 40.6 Hz), 126.8, 120.4, 118.8 (q, *J* = 272.7 Hz), 112.4, 109.4, 13.0,

11.6, 11.5. IR (neat): $\tilde{\nu}$ = 3307, 2923, 2853, 1649, 1522, 1219, 1132, 825, 740, 507 cm^{-1}. HRMS (ESI) *m/z* $(M+H)^+$ calcd for $C_{19}H_{16}F_3N_6O_3$ 433.1231, found 433.1227.

4-Methyl-*N*-(4-(4-(3-methyl-1,2,4-oxadiazol-5-yl)-5-(trifluoromethyl)-1*H*-pyrazol-1-yl)phenyl)-1,2,3-thiadiazole-5-carboxamide, (33).

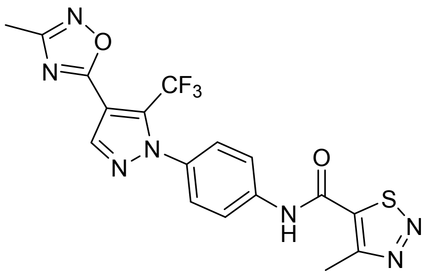

Compound **33** was synthesized following the general procedure starting from compound **17** (100 mg, 0.32 mmol) and 4-methyl-1,2,3-thiadiazole-5-carboxylic acid (50.5 mg, 0.35 mmol). Yellow solid (111 mg, 0.26 mmol, 80%); Chromatography: PE/EtOAc 8:2; mp: 143-144 °C, dec. ^{1}H-NMR (400 MHz; $CDCl_3$): δ 8.28 (s, 1H), 7.82 (d, *J* = 7.4 Hz, 2H), 7.52 (d, *J* = 7.4 Hz, 2H), 2.98 (s, 3H), 2.50 (s, 3H). ^{13}C-NMR (101 MHz; $CDCl_3$): δ 168.5, 167.9, 160.1, 157.6, 143.1, 141.4, 138.5, 135.6, 131.5 (q, *J* = 41.4 Hz), 126.9, 120.8, 116.3 (q, *J* = 272.7 Hz), 109.5, 13.8, 11.6. IR (neat): $\tilde{\nu}$ = 3267, 2927, 2852, 1650, 1519, 1324, 1135, 979, 835, 554 cm^{-1}. HRMS (ESI) *m/z* $(M+H)^+$ calcd for $C_{17}H_{13}F_3N_7O_2S$ 436.0798, found 436.0797.

***N*-(4-(4-(3-methyl-1,2,4-oxadiazol-5-yl)-5-(trifluoromethyl)-1*H*-pyrazol-1-yl)phenyl)furan-2-carboxamide, (34).**

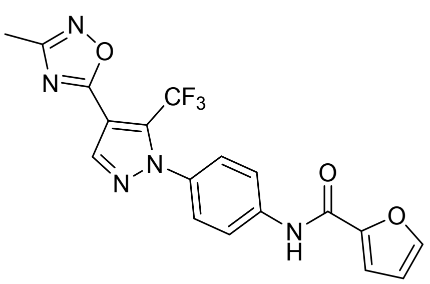

Compound **34** was synthesized following the general procedure starting from compound **17** (100 mg, 0.32 mmol) and furan-2-carboxylic acid (39.2 mg, 0.35 mmol). White solid (70 mg, 0.17 mmol, 54%); Chromatography: PE/EtOAc 8:2; mp: 140-141 °C, ^{1}H-NMR (400 MHz; $CDCl_3$): δ 8.28 (s, 1H), 8.26

(br s, 1H), 7.87 (d, J = 8.8 Hz, 2H), 7.57 (d, J = 1.7 Hz, 1H), 7.50 (d, J = 8.8 Hz, 2H), 7.31 (d, J = 3.5 Hz, 1H), 6.62 (dd, Js = 3.5, 1.7 Hz, 1H), 2.51 (s, 3H). ^{13}C-NMR (101 MHz; $CDCl_3$): δ 168.6, 167.8, 156.1, 147.4, 144.5, 141.3, 139.2, 134.6, 131.4 (q, J = 40.7 Hz), 126.7, 120.0, 119.0, 114.9, 112.8, 109.3, 11.6. IR (neat): $\tilde{\nu}$ = 3395, 3324, 3107, 2921, 2851, 1669, 1630, 1541, 1515, 980 cm^{-1}. HRMS (ESI) *m/z* $(M+H)^+$ calcd for $C_{18}H_{13}F_3N_5O_3$ 404.0965, found 404.0963.

4-Bromo-*N*-(4-(4-(3-methyl-1,2,4-oxadiazol-5-yl)-5-(trifluoromethyl)-1*H*-pyrazol-1-yl)phenyl)-1*H*-pyrrole-2-carboxamide, (35).

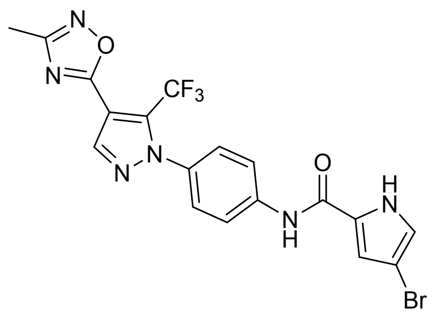

Compound **35** was synthesized following the general procedure starting from compound **17** (100 mg, 0.32 mmol) and 4-bromo-1*H*-pyrrole-2-carboxylic acid (66.5 mg, 0.35 mmol). Yellow solid (85 mg, 0.18 mmol, 55%); Chromatography: PE/EtOAc 8:2; mp: 171-173 °C, dec. ^{1}H-NMR (400 MHz; $(CD_3)_2CO$): δ 11.27 (br s, 1H), 9.56 (br s, 1H), 8.40 (s, 1H), 8.05 (d, J = 8.0 Hz, 2H), 7.61 (d, J = 8.0 Hz, 2H), 7.20 (s, 1 H), 7.13 (s, 1H), 2.45 (s, 3H). ^{13}C-NMR (101 MHz; $(CD_3)_2CO$): δ 168.6, 167.8, 158.2, 141.0, 134.1, 130.8 (q, J = 40.3 Hz), 128.9, 126.6, 124.9, 122.7, 119.8, 119.0 (q, J = 271.7 Hz), 112.6, 108.9, 96.2, 10.5. IR (neat): $\tilde{\nu}$ = 3273, 3094, 3051, 2971, 2926, 2873, 1660, 1631, 978, 834, 802 cm^{-1}. HRMS (ESI) *m/z* $(M+H)^+$ calcd for $C_{18}H_{13}BrF_3N_6O_2$ 481.0230 and 483.0209, found 481.0232 and 483.0211.

Synthesis of 3-(*N*-(2-methoxyphenyl)sulfamoyl)-4-methylbenzoic acid.

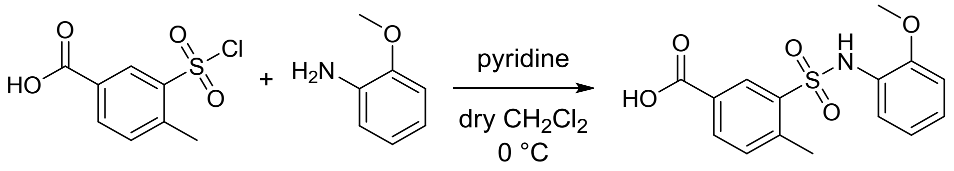

To a solution of 2-methoxyaniline (157 mg, 1.28 mmol) in dry CH_2Cl_2 (4 mL), pyridine (309 μL, 3.84 mmol) and 3-(chlorosulfonyl)-4-methylbenzoic acid (300 mg, 1.28 mmol) were added at 0 °C. After 5 h at 0 °C, the reaction was not concluded and 2-methoxyaniline (0.5 eq) and pyridine (1.5 eq) were added. After stirring for additional 4 h at 0 °C, the reaction was concluded. The mixture was diluted with CH_2Cl_2, washed with HCl 3N (2x) and the organic layer was dried over sodium sulfate and evaporated, affording 3-(*N*-(2-methoxyphenyl)sulfamoyl)-4-methylbenzoic acid (152 mg, 0.47 mmol, 37%) as a pink solid. ^{1}H-NMR (300 MHz; $CDCl_3$): δ 8.62 (s, 1H), 8.08 (d, *J* = 7.7 Hz, 1H), 7.43 (d, *J* = 7.4 Hz, 1H), 7.38 (d, *J* = 7.4 Hz, 1H), 7.01 (d, *J* = 6.8 Hz, 1H), 6.86 (t, *J* = 6.8 Hz, 1H), 6.78 (d, *J* = 6.8 Hz, 1H), 3.77 (s, 3H), 2.74 (s, 3H). HRMS (ESI) *m/z* $(M-H)^-$ calcd for $C_{15}H_{14}NO_5S$ 320.0587, found 320.0593.

Synthesis of 3-(*N*-(2-methoxyphenyl)sulfamoyl)-4-methyl-*N*-(4-(4-(3-methyl-1,2,4-oxadiazol-5-yl)-5-(trifluoromethyl)-1*H*-pyrazol-1-yl)phenyl)benzamide, (36).

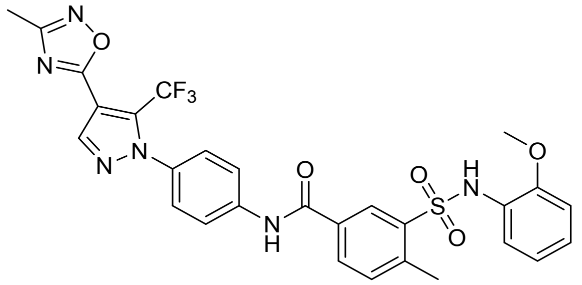

Compound **36** was synthesized following the general procedure starting from compound **17** and 3-(*N*-(2-methoxyphenyl)sulfamoyl)-4-methylbenzoic acid (112 mg, 0.35 mmol).. Purification by column chromatography using PE/EtOAc 7:3 afforded the desired compound **35** as a white solid (129 mg, 0.21 mmol, 66%). Mp: 205-206 °C. ^{1}H-NMR (400 MHz; $(CD_3)_2CO$): δ 10.03 (br s, 1H), 8.47 (s, 1H), 8.39 (s, 1H), 8.30 (br s, 1H), 8.15 (d, *J* = 7.9 Hz, 1H), 8.09 (d, *J* = 6.9 Hz, 2H), 7.62 (d, *J* = 6.9 Hz, 2H), 7.55 (d, *J* = 8.0 Hz, 1H), 7.43 (d, *J* = 7.9 Hz, 1H), 7.09 (t, *J* = 7.9 Hz, 1H), 6.92-6.86 (m, 2H), 3.70 (s, 3H), 2.77 (s, 3H), 2.45 (s, 3H). ^{13}C-NMR (101 MHz; $(CD_3)_2CO$): δ 168.6, 167.8, 164.3, 151.3, 141.6, 141.0, 140.9, 138.9, 134.5, 132.7, 132.6, 131.6, 130.8 (q, *J* = 40.2 Hz), 128.8, 126.6, 126.1, 125.6, 123.1, 120.7, 120.4, 119.7 (q, *J* = 271.6 Hz), 111.2, 109.2, 55.1, 19.5, 10.6. IR (neat):

$\tilde{\nu}$ = 3387, 2922, 2852, 1666, 1518, 1298, 1152, 835, 737, 577 cm^{-1}. HRMS (ESI) *m/z* $(M+H)^+$ calcd for $C_{28}H_{24}F_3N_6O_5S$ 613.1475, found 613.1475.

***N*-(4-(4-(3-Methyl-1,2,4-oxadiazol-5-yl)-5-(trifluoromethyl)-1*H*-pyrazol-1-yl)phenyl)hexanamide, (37).**

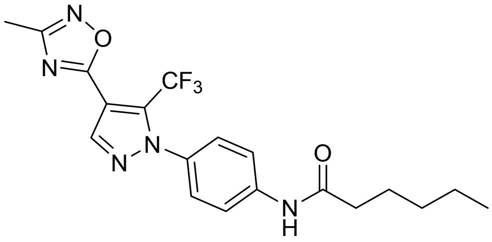

Compound **37** was synthesized following the general procedure starting from compound **17** (100 mg, 0.32 mmol) and hexanoic acid (40.7 mg, 0.35 mmol). White solid (73 mg, 0.18 mmol, 56%); Chromatography: PE/EtOAc 8:2; mp: 176-178 °C, dec. ^{1}H-NMR (400 MHz; $CDCl_3$): δ 8.27 (s, 1H), 7.73 (d, *J* = 8.8 Hz, 2H), 7.45 (d, *J* = 8.8 Hz, 2H), 7.30 (br s, 1H), 2.52 (s, 3H), 2.43 (t, *J* = 7.4 Hz, 2H), 1.79 (quint, *J* = 7.4 Hz, 2H), 1.43-1.39 (m, 4H), 0.94 (t, *J* = 7.4 Hz, 3H). ^{13}C-NMR (101 MHz; $CDCl_3$): δ 171.7, 168.6, 167.8, 141.2, 139.8, 134.3, 131.4 (q, *J* = 40.4 Hz), 126.6, 119.8, 119.0 (q, *J* = 272.7 Hz), 109.3, 37.8, 31.4, 25.2, 22.4, 13.9, 11.6. IR (neat): $\tilde{\nu}$ = 3270, 3092, 2952, 2929, 2866, 1667, 1630, 1519, 1496, 978 cm^{-1}. HRMS (ESI) *m/z* $(M+H)^+$ calcd for $C_{19}H_{21}F_3N_5O_2$ 408.1642, found 408.1640.

***N*-(4-(4-(3-Methyl-1,2,4-oxadiazol-5-yl)-5-(trifluoromethyl)-1*H*-pyrazol-1-yl)phenyl)dodecanamide, (38).**

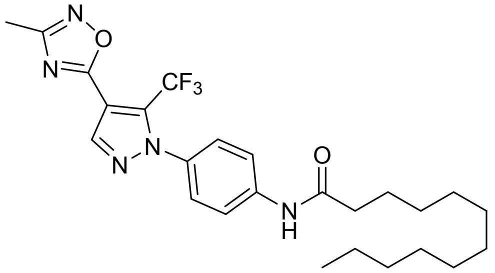

Compound **38** was synthesized following the general procedure starting from compound **17** (100 mg, 0.32 mmol) and dodecanoic acid (70.1 mg, 0.35 mmol). White solid (86 mg, 0.18 mmol, 55%); Chromatography: PE/EtOAc 8:2; mp: 138-140 °C, ^{1}H-NMR (400 MHz; $CDCl_3$): δ 8.26 (s, 1H), 7.73

(d, J = 8.7 Hz, 2H), 7.45-7.43 (m, 3H), 2.51 (s, 3H), 2.42 (t, J = 7.4 Hz, 2H), 1.77 (quint, J = 7.4 Hz, 2H), 1.32-1.29 (m, 16H), 0.90 (t, J = 7.4 Hz, 3H). ^{13}C-NMR (101 MHz; $CDCl_3$): δ 172.1, 171.8, 167.8, 141.2, 139.9, 131.4 (q, J = 40.9 Hz), 127.0, 126.5, 119.7, 118.9 (q, J = 272.6 Hz), 109.2, 46.7, 37.8, 34.9, 31.9, 29.6, 26.1, 25.5, 25.0, 24.4, 22.7, 14.1, 11.6. IR (neat): $\tilde{\nu}$ = 3278, 3096, 2918, 2848, 1629, 1522, 1466, 1134, 979, 842 cm^{-1}. HRMS (ESI) *m/z* $(M+H)^+$ calcd for $C_{25}H_{33}F_3N_5O_2$ 492.2581, found 492.2577.

***N*-(4-(4-(3-Methyl-1,2,4-oxadiazol-5-yl)-5-(trifluoromethyl)-1*H*-pyrazol-1-yl)phenyl)-3-morpholinopropanamide, (39).**

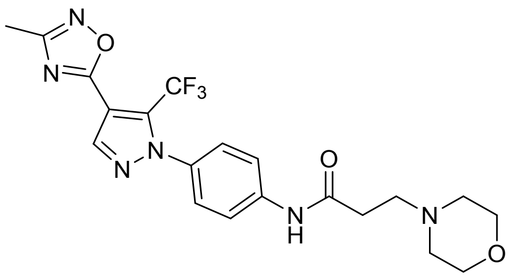

Compound **39** was synthesized following the general procedure starting from compound **17** (100 mg, 0.32 mmol) and 3-morpholinopropanoic acid (55.7 mg, 0.35 mmol). White solid (58 mg, 0.13 mmol, 40%); Chromatography: EtOAc; mp: 147-149 °C. ^{1}H-NMR (400 MHz; $CDCl_3$): δ 8.26 (s, 1H), 7.75 (d, J = 8.8 Hz, 2H), 7.44 (d, J = 8.8 Hz, 2H), 6.37 (br s, 1H), 3.90 (t, J = 4.4 Hz, 4H), 2.91 (t, J = 5.4 Hz, 2H), 2.78-2.75 (m, 4H), 2.70 (t, J = 5.4 Hz, 2H), 2.51 (s, 3H). ^{13}C-NMR (101 MHz; $CDCl_3$): δ 170.2, 168.6, 167.8, 141.2, 140.2, 134.1, 131.4 (q, J = 40.7 Hz), 126.6, 120.4, 118.9 (q, J = 272.5 Hz), 109.2, 66.6, 54.0, 52.8, 32.0, 11.6. IR (neat): $\tilde{\nu}$ = 3275, 3090, 2921, 2851, 1659, 1519, 1133, 977, 722, 703 cm^{-1}. HRMS (ESI) *m/z* $(M+H)^+$ calcd for $C_{20}H_{22}F_3N_6O_3$ 451.1700, found 451.1695.

***N*-(4-(4-(3-Methyl-1,2,4-oxadiazol-5-yl)-5-(trifluoromethyl)-1*H*-pyrazol-1-yl)phenyl)tetrahydro-2*H*-thiopyran-4-carboxamide 1,1-dioxide, (40).**

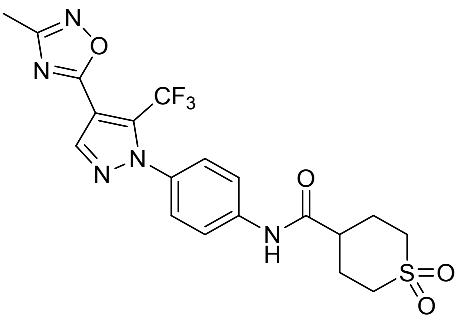

Compound **40** was synthesized following the general procedure starting from compound **17** (100 mg, 0.32 mmol) and tetrahydro-2*H*-thiopyran-4-carboxylic acid 1,1-dioxide (62.4 mg, 0.35 mmol). White solid (63 mg, 0.13 mmol, 42%); Chromatography: PE/EtOAc 3:7; mp: 153.5-154.5 °C, ^{1}H-NMR (400 MHz; DMSO-d_6): δ 10.38 (br s, 1H), 8.55 (s, 1H), 7.82 (d, J = 8.9 Hz, 2H), 7.55 (d, J = 8.9 Hz, 2H), 3.29-3.19 (m, 4H), 2.75-2.77 (m, 1H), 2.23 (s, 3H), 2.21-2.18 (m, 2H), 2.15-2.09 (m, 2H). ^{13}C-NMR (101 MHz; DMSO-d_6): δ 172.9, 168.6, 168.2, 141.7, 141.2, 133.7, 130.7 (q, J = 39.8 Hz), 127.2, 119.9, 119.4 (q, J = 272.2 Hz), 108.9, 49.9, 41.5, 27.5, 11.6. IR (neat): $\tilde{\nu}$ = 3301, 3092, 2947, 2929, 1649, 1633, 1523, 1323, 1143, 977 cm^{-1}. HRMS (ESI) *m/z* (M+H)$^+$ calcd for $C_{19}H_{19}F_3N_5O_4S$ 470.1104, found 470.1106.

***N*-(4-(4-(3-Methyl-1,2,4-oxadiazol-5-yl)-5-(trifluoromethyl)-1*H*-pyrazol-1-yl)phenyl)-2-phenoxyacetamide, (41).**

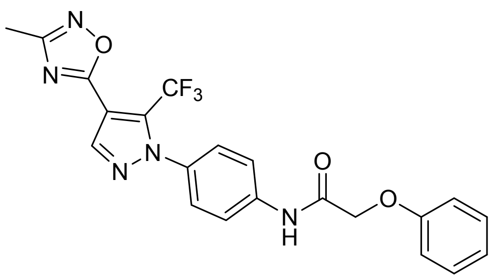

Compound **41** was synthesized following the general procedure starting from compound **17** (100 mg, 0.32 mmol) and 2-phenoxyacetic acid (53.3 mg, 0.35 mmol). White solid (86 mg, 0.19 mmol, 61%); Chromatography: PE/EtOAc 7:3; mp: 160-161 °C, dec. ^{1}H-NMR (400 MHz; $CDCl_3$): δ 8.48 (br s, 1H), 8.28 (s, 1H), 7.82 (d, J = 8.8 Hz, 2H), 7.50 (d, J = 8.8 Hz, 2H), 7.40 (t, J = 7.6 Hz, 2H), 7.11 (t, J = 7.6 Hz, 1H), 7.04 (d, J = 7.6 Hz, 2H), 4.69 (s, 2H), 2.52 (s, 3H). ^{13}C-NMR (101 MHz; $CDCl_3$): δ 168.6, 167.8, 166.6, 156.9, 141.3, 138.6, 135.0, 131.4 (q, J = 40.7 Hz), 130.0, 126.7, 122.7, 120.2,

119.0 (q, J = 272.4 Hz), 114.9, 109.4, 42.8, 10.6. IR (neat): $\tilde{\nu}$ = 3356, 3103, 3087, 2922, 2903, 1674, 1631, 1229, 1128, 982 cm^{-1}. HRMS (ESI) *m/z* $(M+H)^+$ calcd for $C_{21}H_{17}F_3N_5O_3$ 444.1278, found 444.1280.

Synthesis of 4-methyl-*N*-(4-(4-(3-methyl-1,2,4-oxadiazol-5-yl)-5-(trifluoromethyl)-1*H*-pyrazol-1-yl)phenyl)benzenesulfonamide, (42).

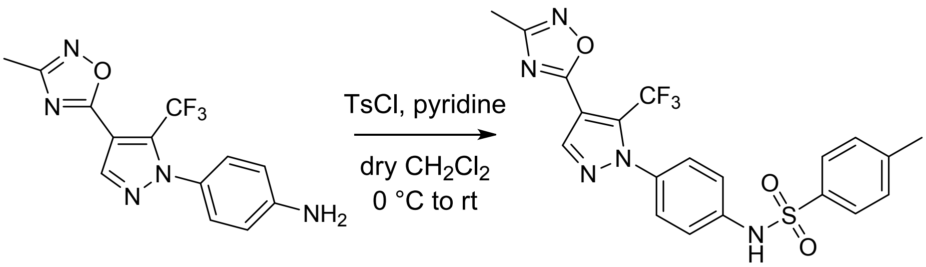

4-(4-(3-Methyl-1,2,4-oxadiazol-5-yl)-5-(trifluoromethyl)-1*H*-pyrazol-1-yl)aniline **17** (100 mg, 0.32 mmol) was solubilized in dry CH_2Cl_2 (2.5 mL) and pyridine (77 µL, 0.96 mmol) and *p*-toluenesulfonyl chloride (TsCl) (72 mg, 0.38 mmol) were added in order at 0 °C. The mixture was allowed to reach rt and stirred for 5 h. Then, the reaction was diluted with CH_2Cl_2, washed with HCl 3N (2x), dried over sodium sulfate and evaporated. Purification by column chromatography using PE/EtOAc 7:3 as eluent afforded the desired compound **42** (67 mg, 0.14 mmol, 45%) as a yellow solid. Mp: 149-150 °C. ^{1}H-NMR (400 MHz; $CDCl_3$): δ 8.25 (s, 1H), 7.75 (d, J = 7.4 Hz, 2H), 7.54 (br s, 1H), 7.36 (d, J = 7.9 Hz, 2H), 7.29-7.26 (m, 4H), 2.50 (s, 3H), 2.41 (s, 3H). ^{13}C-NMR (101 MHz; $CDCl_3$): δ 168.5, 167.9, 144.5, 141.3, 138.6, 135.7, 135.3, 131.4 (q, J = 40.4 Hz), 129.9, 127.3,, 127.0, 120.9, 118.9 (q, J = 272.7 Hz), 109.4, 21.5, 11.6. IR (neat): $\tilde{\nu}$ = 3173, 2922, 2852, 1631, 1514, 1324, 1147, 919, 809, 541 cm^{-1}. HRMS (ESI) *m/z* $(M+H)^+$ calcd for $C_{20}H_{17}F_3N_5O_3S$ 464.0999, found 464.0998.

***In vitro* metabolism and purity of lead compounds**

In vitro metabolism studies

Mouse liver S9 (MLS9) (pooled male mouse CD-1, protein concentration: 20 mg/mL) were purchased from Corning B.V. Life Sciences (Amsterdam, The Netherlands) and used throughout this study. The standard incubation mixture (250 μL final volume) was carried out in a 50 mM TRIS (tris[hydroxymethyl]-aminomethane) + 150 mM KCl buffer (pH 7.4) containing 3.3 mM $MgCl_2$, 1.3 mM β-NADP-Na_2, 3.3 mM glucose 6-phosphate, 0.4 Units/mL glucose 6-phosphate dehydrogenase (NADPH regenerating system), 5 μL of acetonitrile (1% of total volume), and the substrate compounds at a concentration of 50 μM. After pre-equilibration of the mixture, an appropriate volume of MLS9 suspension was added to give a final protein concentration of 1.5 mg/mL. The mixture was shaken for 60 min at 37 °C. Each incubation was stopped by addition of 250 μL of ice-cold acetonitrile, vortexed, and centrifuged at 13000 rpm for 10 min. Control incubations were carried out without the presence of MLS9 suspension or cofactors or in absence of the substrate.

Chromatographic method

LC-UV method for purity and metabolic stability data determination: a Shimadzu HPLC system (Shimadzu, Kyoto, Japan), consisting of two LC-10AD Vp module pumps, an SLC-10A Vp system controller, an SIL-10AD Vp autosampler, and a DGU-14-A on-line degasser were used for the analysis. The SPD-M10Avp photodiode array detector was used to detect the analytes. LC-Solution 1.24 software was used to process the chromatograms.

- Column: *Phenomenex Kinetex XB C18, 150 × 4.6 mm (5 μm d.p.)* protected with a SecurityGuard® (Torrance, CA, USA).
- Eluant:

  A: 0.2% formic acid in water;

  B: 0.2% formic acid in acetonitrile.
- Flow rate: 1 mL/min.

- Injection volume: 20 μL.
- Wavelength: 265 nm for **22**, **27**, **32**, **37**, **42**, and 285 nm for **29**, **23**
- Gradient program: 0.00 min [B%=30%], 10.00 min [B%=90%], 12.50 min [B%=90%], 13.00 min [B%=30%], 18.00 min [B%=30%].

Biology

Cell culture

Human embryonic kidney (Hek) cells were obtained from ATCC (Rockville, MD, USA) and were cultured in Dulbecco's Modified Eagle's Medium (DMEM; Sigma-Aldrich, Italy), supplemented with 10% heat-inactivated FBS (Gibco, Italy), L-glutamine 50 mg/mL (Sigma-Aldrich, Italy), 10 U/mL penicillin, and 100 mg/mL streptomycin (Sigma-Aldrich, Italy) at 37 °C, under a 5% CO_2 humidified atmosphere. For calcium experiments, cells were plated onto glass coverslips at concentrations 5×10^4 per mL (24 mm diameter coverslips in 6-well plates) or 3×10^4 per mL in 24-well plates.

Fluo-4 Ca^{2+} Measurements

For a preliminary screening, 3V Victor plate reader (PerkinElmer, USA) was used, allowing the use of 24-well. Briefly, Hek cells were loaded with Fluo-4 AM 2.5 μM in the presence of 0.02% of Pluronic-127 (both from Life Technologies, Italy) and 10 μM sulfinpyrazone in Krebs−Ringer buffer (KRB, 135 mM NaCl, 5 mM KCl, 0.4 mM KH_2PO_4, 1 mM $MgSO_4$, 5.5 mM glucose, 20mM HEPES, pH 7.4) containing 2 mM $CaCl_2$ (30 min, room temperature). After which, cells were washed and incubated with KRB for other 30 min to allow de-esterification of Fluo-4 AM. To measure SOCE, cells were depleted of calcium in the endoplasmic reticulum with 2,5-t-butylhydroquinone (*t*BhQ, 50 μM; Sigma-Aldrich, Italy), a SERCA poison, in a Ca^{2+}-free solution, and Ca^{2+} was re-added to the extracellular solution. Fluorescence was then monitored sequentially for a second in each well for 600 s, generating time curves for each well.

Fura-2 Ca^{2+} Measurements

For calcium imaging experiments, Hek cells were plated onto pre-treated poli-L-lysine (P4832, Sigma-Aldrich, Italy) glass coverslips of 24 mm in 6-well plates Cells were loaded with 5 μM of Fura-2 AM, 0.02% of Pluronic-127 (Life technologies, Italy) and 10 μM of sulfinpyrazone (Sigma-Aldrich, Italy) in KRB containing 2 mM $CaCl_2$ for 30 min at RT. Cells were then washed once with KRB for 15 min for de-esterification of Fura-2 AM.

Coverslips were mounted into the acquisition chamber and placed on the stage of a Leica DMI6000 epifluorescent microscope equipped with S Fluor ×40/1.3 objective. Fura-2 AM was excited by alternate 340 and 380 nm using a Polychrome IV monochromator (Till Photonics, Germany), and the probe emission light was filtered through 520/20 bandpass filter and collected by a cooled CCD camera (Hamamatsu, Japan). The fluorescence signals were acquired and processed using MetaFluor software (Molecular Device, Sunnyvale, CA, USA). To quantify the differences in the amplitudes of Ca^{2+} transients, the ratio values were normalized using the formula ΔF/F0.

3-(4,5-Dimethylthiazol-2-yl)-2,5-diphenyltetrazolium bromide (MTT) assay

Hek cells were plated in 24-wells plate at 20.000 cells per well. After 24 h, cells were treated for other 24 h with the selected compounds. At the end of treatments, medium was removed and substituted by 300 µL of MTT reagent at the final concentration of 0.25 mg/mL for 60 min at 37 °C. Reactions were then stopped and the crystals were solubilized by adding isopropyl alcohol/HCl (1:1; vol:vol) (Sigma-Aldrich Inc., Italy), before reading the absorbance at 570 nm, using the multi-plate reader Victor 3V (PerkinElmer, Milan, Italy).

Statistical analysis

In calcium imaging experiments, data are presented as Mean +/- standard error (SEM) or Median and interquartile range (IQR). The normality of data distributions was assessed using the Shapiro−Wilk test. Nonparametric (Mann−Whitney U test and one-way) statistical analysis was used for comparisons of data. All statistical assessments were two-sided and a value of P <0.05 was considered statistically significant. Statistical analyses were performed using GraphPad Prism software (GraphPad Software, Inc., USA).

References

(1) *a)* Riva, B.; Griglio, A.; Serafini, M.; Cordero-Sanchez, C.; Aprile, S.; Di Paola, R.; Gugliandolo, E.; Alansary, D.; Biocotino, I.; Lim, D.; Grosa, G.; Galli, U.; Niemeyer, B.; Sorba, G.; Canonico, P. L.; Cuzzocrea, S.; Genazzani, A. A.; Pirali, T. Pyrtriazoles, a novel class of store-operated calcium entry modulators: discovery, biological profiling, and *in vivo* proof-of-concept efficacy in acute pancreatitis. *J. Med. Chem.* **2018**, *61* (21), 9756-9783; *b)* Pirali, T.; Riva, B.; Genazzani, A. A. Modulators of SOCE, compositions and use thereof. W.O. Patent 212,414, Dec 14, 2017.
